# Neural excitation/inhibition imbalance and neurodevelopmental pathology in human copy number variant syndromes: a systematic review

**DOI:** 10.1186/s11689-025-09614-8

**Published:** 2025-06-09

**Authors:** Amy L. Sylvester, Eva Hensenne, Dimo Ivanov, Benedikt A. Poser, David E. J. Linden, Thérèse van Amelsvoort, Claudia Vingerhoets

**Affiliations:** 1 Department of Psychiatry and Neuropsychology, Mental Health and Neuroscience Research Institute, Maastricht University, Maastricht, Netherlands; 2 Department of Cognitive Neuroscience, Faculty of Psychology and Neuroscience, Maastricht University, Maastricht, Netherlands; 3 Max Planck Institute for Empirical Aesthetics, Frankfurt, Germany; 4 Cooperative Brain Imaging Center - CoBIC, Frankfurt, Germany; 5 Advisium ’s Heeren Loo Zorggroep, Amersfoort, Netherlands

**Keywords:** Neurodevelopmental disorders, Copy number variation, Excitation, Inhibition, Glutamate, γ-aminobutyric acid

## Abstract

Cumulative evidence suggests neurodevelopmental disorders are closely related. The risk of these disorders is increased by a series of copy number variant syndromes – phenotypically heterogeneous genetic disorders, present in a minority of the population. Recent models suggest that a disruption in the balance between excitatory and inhibitory neural activity may contribute to the aetiology of neurodevelopmental disorders, and may be additionally disturbed in copy number variant syndromes. In this systematic review, the databases PubMed, Embase, and Scopus were searched for studies of excitation/inhibition imbalance in relation to neurodevelopmental disorders in human copy number variant samples. A total of 53 studies were included, representing a variety of copy number variants and research methodologies. The resulting data suggests excitation/inhibition balance is indeed disrupted in different copy number variant populations, providing insight into a putative mechanism of both idiopathic and genetic neurodevelopmental disorders. However, the high level of heterogeneity in the data set, alongside emerging techniques for excitation/inhibition assessment, prompts further investigation of this field.

**Supplementary Information:**

The online version contains supplementary material available at 10.1186/s11689-025-09614-8.

## Background

### Neurodevelopmental disorders

Neurodevelopmental disorders (NDD) historically refer to psychiatric conditions manifesting in childhood, including intellectual disability (ID), autism spectrum disorder (ASD), and attention deficit hyperactivity disorder (ADHD). Accumulating evidence has led many to suggest that the late-adolescence- and adult-onset psychiatric disorders schizophrenia (SCZ) [1–4] and bipolar disorder (BD) [5, 6] might also be classified as neurodevelopmental. Neurodevelopmental hypotheses of mental disorders state neural alterations resulting from genetic and environmental factors early in development interact with or disrupt normal brain maturation over the course of childhood, adolescence, and early adulthood, leading to psychiatric symptoms. NDDs share phenotypic similarities (for example cognitive impairment), are highly comorbid with one another, have shared environmental risk factors, and have a high degree of common genetic risk [7, 8]. There is also a significant unmet clinical need for effective treatment in NDD populations [9], highlighting the urgency of uncovering new therapeutic mechanisms.

### Genetic factors in neurodevelopmental disorders

Neurodevelopmental disorders are known to have a strong genetic basis. All are highly heritable, but seldom have a single causative factor. Genome-wide investigations of genetic factors in NDDs have revealed a constellation of common single nucleotide polymorphisms (SNPs) associated with each NDD [10]. Of note is the high degree of overlap between the individual risk profiles of each NDD, and the predominance of genes associated with synaptic transmission [11]. This is consistent with phenotypic commonalities between disorders. However, the odds ratio conveyed by any one of these risk polymorphisms is small. A much higher genetic risk is instead conveyed by rare anomalies which can be inherited but are more often acquired de novo: small single nucleotide variants (SNVs) and large, recurrent copy number variants (CNVs) [12, 13]. CNVs are defined as regions of deletion, duplication, translocation, or inversion that are larger than 100-thousand base pairs (100kb) and present in less than 1% of the human population [14]. CNVs such as the 22q11.2 deletion, 2p16.3 deletion, 3q29 deletion, and 16p11.2 duplication, are among the strongest genetic risk factors for schizophrenia [15, 16]. Other CNVs are particularly prevalent in other NDDs, for example, 15q11-13 duplication and 16p11.2 deletion in both ASD and ADHD [17, 18]. The inverse is also true: the rate of CNVs is increased in NDD cases compared to controls [14, 19]. Crucially however, each of the CNVs mentioned above have a highly heterogenous phenotype. These and some other, rarer variants are associated with drastically increased risk of multiple NDDs, and particularly cognitive impairment and intellectual disability. As a result, CNV populations are an important target group for the investigation of NDD pathophysiology.

### Excitation/inhibition imbalance

Whilst proposed mechanisms of NDDs are many and varied, disruption of the excitation/inhibition (E/I) balance in the brain is applicable to many of the most common NDD-associated CNV syndromes. E/I balance refers to the ratio of excitatory to inhibitory neural activity, and consequentially the ratio of the primary excitatory and inhibitory neurotransmitters: glutamate and γ-aminobutyric acid (GABA) respectively [20]. The balance of excitatory to inhibitory activity is intrinsically linked to neurodevelopment. Neural circuitry matures in “critical periods” of neurodevelopment, from primary sensory-motor cortices in early childhood to higher-order cognitive cortices in adolescence [21]. The prefrontal cortex, crucial to the pathology of SCZ, is among the last brain regions to mature [22]. Regulation of these critical periods is thought to depend on maturation of GABAergic inhibitory circuitry [23], and as inhibitory control increases throughout neurodevelopment, the cortical E/I ratio decreases [24, 25]. Disruption of E/I balance during these critical periods of development is then associated with the onset of neurodevelopmental disorders [26].

The evidence for E/I imbalance in ASD and SCZ is particularly compelling, and has been reviewed extensively elsewhere [27–32]. In brief, E/I abnormalities in idiopathic NDDs have been widely documented, from the molecular level, where brain glutamatergic metabolite levels are significantly more variable in SCZ patients compared to controls [33] and higher glutamate levels predict increased psychotic symptom severity [34], to the whole-brain network level, where electroencephalographic gamma-band power, thought to reflect excitatory connectivity, is consistently increased in ASD [35]. Indications are also present in ADHD and BD [36–38]. In ADHD and BD too, the ratio of GABA to glutamate appears to be reduced and correlates with impaired inhibitory control and executive function [39–41].

Notably, affected regions in CNVs known to be associated with NDDs have been found to contain and have downstream effects on genes linked to excitatory and inhibitory synapses. For example, chromosomal region 15q11.2-q13.1, the critical region in 15q11-13 duplication syndrome and in Angelman and Prader-Willi syndromes contains three GABA receptor genes (*GABRB3*, *GABRA5*, and *GABRG3*). The 22q11.2 locus includes proline dehydrogenase (*PRODH*), a mitochondrial enzyme that converts proline to glutamate. As well as being directly involved in glutamate metabolism, proline itself also acts as a co-agonist of the glutamatergic NMDA receptor [42]. Also within the 22q11.2 deletion locus is the transcription factor *TBX1*, the loss of which has been demonstrated to alter the development of glutamatergic cortical projection neurons and GABAergic interneurons in the mouse neocortex [43]. Additionally, E/I-related genes have been found to be enriched in CNV association studies of NDDs [44, 45], as well as wider genetic association studies [46]. The extent and nature of E/I imbalance is likely to vary with the mutation size and implicated genes in any given CNV syndrome. However, E/I imbalance as a result of copy number variation may be a key mechanism for the increased propensity towards NDDs in these high-risk groups, and perhaps mirror established E/I imbalance in idiopathic NDDs. Therefore, targeting E/I imbalance could have potential as a therapeutic target, both in CNV carriers and in the wider clinical population.

### Aims and scope

This article will review evidence of E/I imbalance from human carriers of recurrent copy number variants and discuss how these findings may relate to known mechanisms of neurodevelopmental pathology. Whilst significant progress has been made in our understanding of these rare genetic disorders, this review will demonstrate areas of inconsistency and uncertainty and suggest avenues of future study.

## Methods

The review protocol is registered in the International Prospective Register of Systematic Reviews (PROSPERO; CRD42023478130) and was conducted in accordance with the Peer Review of Electronic Search Strategies (PRESS) and Preferred Reporting Items for Systematic reviews and Meta-Analyses (PRISMA) guidelines.

### Search strategy

PubMed, Embase, and Scopus databases were searched from inception to 31st January 2024 to identify articles relating to the following search concepts: (i) Copy number variant syndromes AND (ii) Excitation/inhibition imbalance AND (iii) Neurodevelopmental disorders (ID, ASD, ADHD, SCZ, BD). Embase was filtered to remove non-original publications, due to the high volume of retrieved articles. The reference lists and citations of included articles were hand-searched to identify studies omitted from the electronic search. The full search strategy can be found in Supplement 1.

### Study screening

Retrieved articles were deduplicated in EndNote 20 [47]. Two researchers (AS and EH) independently screened the title and abstract of retrieved articles against eligibility criteria, using Rayyan. Where an inclusion criterion was present only in the key words of an article, the methods section of the article was also reviewed. When necessary, conflicts were resolved by consensus. The full-text of the initially included articles was then assessed against inclusion/exclusion criteria by AS. Inclusion criteria were original peer-reviewed articles, published in English, reporting a measure of excitation/inhibition balance in human participants or cell lines carrying a confirmed recurrent copy number variant associated with neurodevelopmental disorders. Reviews, meta-analyses, case reports, non-human research, studies unrelated to a listed neurodevelopmental disorder, studies that did not experimentally assess excitation/inhibition-related factors, and studies that did not include a CNV sample were excluded.

### Data extraction

Data extraction was conducted by AS, using an adapted form of the Cochrane Consumers and Communication Group Data extraction template [48]. The following information was extracted: (i) General information – authors, year of publication, title, journal, DOI, funder(s); (ii) Methods – study design, aims, informed consent documented, ethical approval documented, CNV studied, neurodevelopmental disorder studied, method of excitation/inhibition balance assessment, randomisation/blinding (where applicable); (iii) Participants – research location, participant setting, recruitment method, inclusion criteria, exclusion criteria, age, gender, ethnicity, initial number in each group, number included in analysis (by group and outcome), reasons for withdrawal; (iv) Study characteristics – CNV diagnostic method, diagnostic/symptom assessment of neurodevelopmental disorder, excitation/inhibition assessment type, experimental procedure, modifications/adaptations; (v) Outcomes – relevant study results.

### Study quality and risk of bias assessment

Study quality and risk of bias was assessed by study type using the Study Quality Assessment Tools of the National Heart, Lung, and Blood Institute of the National Institutes for Health (https://www.nhlbi.nih.gov/health-topics/study-quality-assessment-tools).

## Results

**Fig. 1:**
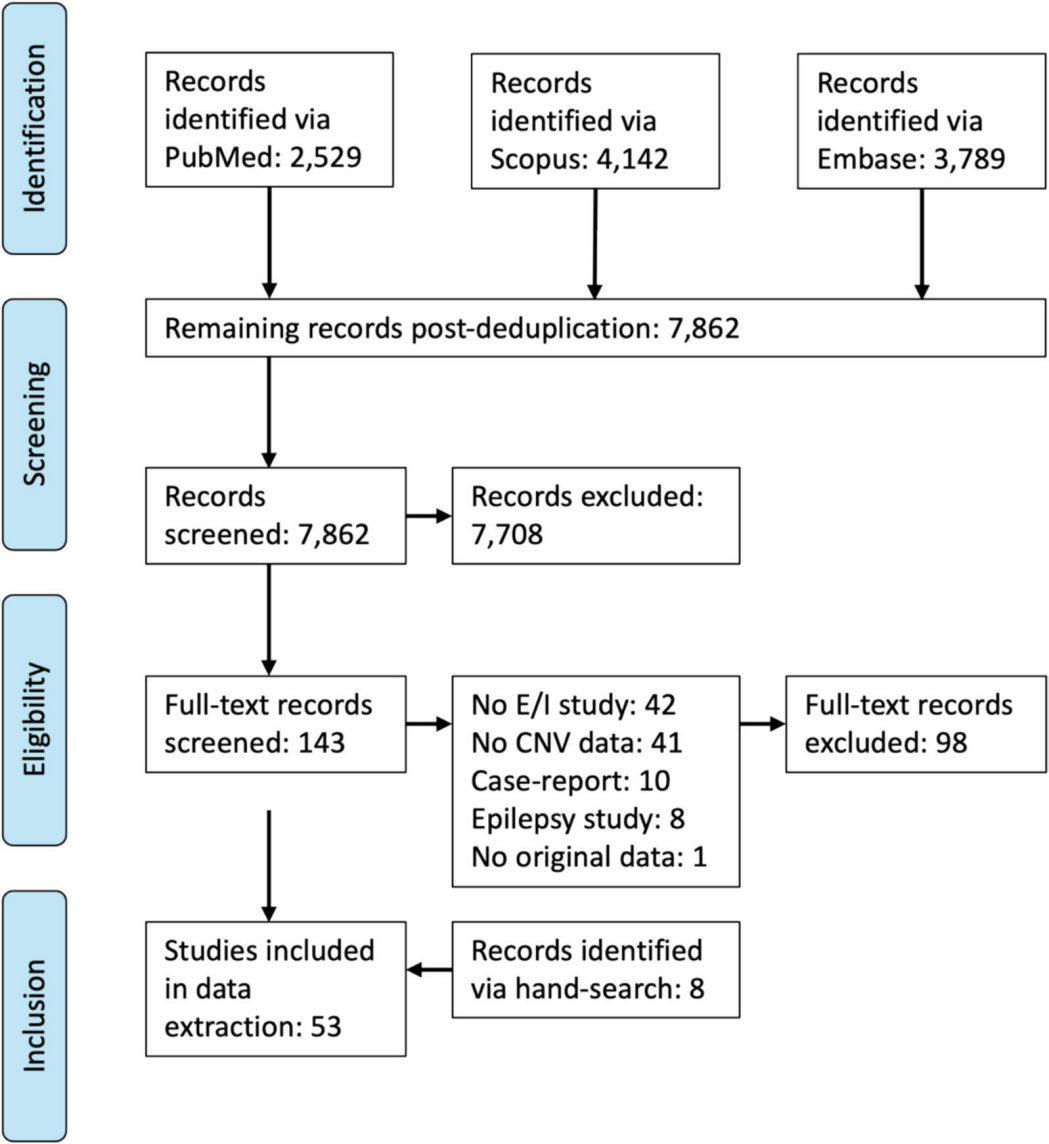
PRISMA flow diagram of study screening and inclusion. Some full-text screened papers met multiple exclusion criteria

The PRISMA flow-chart in Fig. 1 summarises the search and selection process. Fifty-three studies were included from database and hand searching. The included studies are heterogeneous, covering a wide range of E/I assessment modalities, CNV syndromes (described in Table 1), and participant characteristics. Results were organised along four lines of evidence: neurogenomics, neurochemistry, neurophysiology, and neuromodulation, and are summarised in Table 2.

**Table 1:** Overview of represented copy number variant syndromes

Locus	Other name(s)	Size	Incidence	Associated NDD(s)	Genes of interest^(a)^
2p16.3 deletion	NRXN1 deletion	28-806 kb [49]	1/4,000 [49]	ADHD, ASD, ID, SCZ	*NRXN1*
7q11.23 deletion	Williams-Beuren syndrome	1.5–1.8 Mb [50]	1/7,500–10,000 [50]	ADHD, ASD, ID	*HIP1*, *GTF21*, *GTF21RD1*, *LIMK1* [51]
7q11.23 duplication	Dup7; 7dup	1.5–1.8 Mb [50]	1/7,500–20,000^b^ [52]	ADHD, ASD, ID	*HIP1*, *GTF21*, *GTF21RD1*, *LIMK1* [51]
9p24.2 deletion	9p minus; Monosomy 9p^c^	10-500 kb [53]	1/50,000^c^ [54]	ID, SCZ	*SLC1A1* [53]
15q11-13 deletion (maternal)	Angelman syndrome^d^	5.0–5.9 Mb [55]	1/10,000–20,000 [55]	ASD. ID	*GABRB3*, *GABRA5*, *GABRG3*, *UBE3A* [55]
15q11-13 deletion (paternal)	Prader-Willi syndrome^d^	5.0–6.0 Mb [56]	1/10,000–30,000 [56]	ID	*GABRB3*, *GABRA5*, *GABRG3*, *UBE3A* [56]
15q11-13 duplication	Dup15q	5.0–8.7 Mb [57]	1/14,500 [57]	ASD, ID, SCZ	*GABRB3*, *GABRA5*, *GABRG3*, *UBE3A* [58]
16p11.2 deletion	None	600 kb [59]	1/2,000–4,000 [59]	ADHD, ASD, ID	*MAPK3*, *KCTD13*, *TAOK2* [60]
16p11.2 duplication	None	600 kb [59]	1/1,500–2,500 [59]	ADHD, ASD, BD, ID, SCZ	*MAPK3*, *KCTD13*, *TAOK2* [60]
22q11.2 deletion	DiGeorge syndrome, Velocardiofacial syndrome	1.5–3.0 Mb [61]	1/2000 [62]	ADHD, ASD, ID, SCZ	*PRODH*, *COMT* [61]

*Kb* kilobase pairs, *Mb* megabase pairs

^a^Other sources may give additional genes of interest

^b^Estimate based on 7q11.23 deletion prevalence, duplication prevalence not separately estimated

^c^9p minus and Monosomy 9p also refer to other CNVs on chromosome 9

^d^Only a subset of Angelman (~ 70%) and Prader-Willi syndrome (65–75%) individuals carry a deletion. Many genes within this region are subject to imprinting: an epigenetic mechanism whereby genes on the maternally or paternally inherited chromosome are silenced. If a deletion, uniparental disomy (inheritance of both copies of chromosome 15 from a single parent), or other loss-off-function mutation occurs within this region, this leads to Angelman or Prader-Willi syndrome

**Table 2:** Summary of included studies

Excitation/inhibition measure	Study	CNV	Participants Group: N	Age	Outcomes
*Neurogenomics*					
Gene expression	Bittel et al. 2007 [63]	Prader-Willi	In vivo – Prader-Willi deletion: 4 In vivo – Prader-Willi uniparental disomy In vivo – Obese Controls: 3 Post-mortem – carriers: 3 Post-mortem – controls: 3	Mean Prader-Willi deletion (in vivo): 23 years (range: 16–34) Mean Prader-Willi uniparental disomy (in vivo): 29 years (range: 13–45) Mean obese controls (in vivo): 18 years (range: 11–23) Mean carriers (post-mortem):21.67 years (range: 1–32) Mean controls (post-mortem): 37 years (range: 1–71)	Increased expression of GRIN2B, GABRA4, and GABRG2 in Prader-Willi syndrome lymphoblast cultures microarray relative to controls, and decreased expression of GABRA2 and GABRD expression relative to controls. Additional disruption in serotonergic and glycinergic receptors expression. Overall change of expression in 14 genes related neurological development and function
	Hogart et al. 2009 [64]	Prader-Willi; 15q11-13 duplication	Post-mortem – 15q11-13 duplication: 2 Post-mortem – Prader Willi: unknown Post-mortem – Autistic controls: unknown	15q11-13 duplication carriers: 11 and 26 years at time of death. Other groups unknown	Reduced GABRB3 expression in Prader-Willi syndrome post-mortem brains relative to controls. The 15q11-13 duplication post-mortem samples showed differing trajectories of GABA receptor gene expression (elevated in one and decreased in the other)
	Roden et al. 2010 [65]	Angelman	Post-mortem – Carriers: 4 Post-mortem – Unaffected controls: 4	Unknown	Elevated expression of the GABAa receptor β2 subunit, while expression of the ɑ5 subunit was reduced in Angelman syndrome samples relative to controls. The ratio of the β3 to β2, and ɑ5 to ɑ1 subunit expression was reduced in the Angelman syndrome cortex samples. GABAa current enhancement by zoldipem (a benzodiazepine agonist) and phenobarbital increased in Angelman syndrome relative to controls
	Samaco et al. 2005 [66]	Angelman	Post-mortem – Carriers: 2 Post-mortem – Rett syndrome: 3 Post-mortem – ASD controls: 9 Post-mortem – Unaffected controls: 11	Unknown	Angelman syndrome samples showed reduced GABRB3 expression relative to healthy controls, in line with the pattern seen in autistic controls
mRNA expression and transcriptomics	Afshari et al. 2015 [53]	9p24.2 deletion	Carriers: 8 Unaffected relatives: 11	Mean carriers: 55.1 years (range: 20–103) Mean unaffected relatives: 49.6 years (range: 28–73)	Increased expression of GRIA1, GRM8, GRIN2A, and glutamate transporters in deletion carriers relative to controls. Increased expression of GRIK4 in deletion carriers with psychosis relative to carriers without psychosis. Expression of GABBR1, GABRA4, and GABRG2 reflected induced expression of deleted SLC1A1gene
	Scoles et al. 2011 [67]	15q11-13 duplication	Post-mortem – Carriers: 8 Post-mortem – ASD controls: 10 Post-mortem – Unaffected controls: 21	Unknown	Increased variability in 15q11-13 duplication carrier GABRB3 transcript levels, both higher and lower levels than controls
*Neurochemistry*					
Plasma GABA	Borgatti et al. 2001 [68]	15q11-13 duplication	Carriers: 6 Controls: 8	Mean carriers: 8.83 years (range: 4–14) Controls age-matched, specifics unknown	No significant difference in plasma GABA or diazepam binding inhibitor between children with 15q11-13 duplication and healthy control children
	Borgatti et al. 2003 [69]	Angelman	Carriers: 12 Epileptic controls: 8 Healthy controls: 12	Mean carriers: 12.7 years (range: 2–29) Mean epileptic controls: 15.1 years (range: 11–20) Mean healthy controls: 12 years (range: 6–18)	Increased plasma GABA and diazepam binding inhibitor in Angelman syndrome relative to non-epileptic controls but decreased plasma GABA relative to medication-responsive epileptic controls
	Ebert et al. 1997 [70]	Angelman; Prader-Willi	Angelman: 9 Prader-Willi: 14 Obese controls: 7 Non-syndromic ID controls: 5	Range Prader-Willi: 2–21 years Range Angelman: 2–17 years Range obese controls: 2–20 years Range ID controls: range: 3–17 years	Increased mean plasma GABA in Angelman syndrome and Prader-Willi syndrome relative to both control groups. No significant difference between carrier groups. No relationship between GABA levels and deletion presence
Plasma glutamate	Evers et al. 2015 [71]	22q11.2 deletion	Carriers: 64	Mean: 33.7 years (range:18–59)	Hyperprolinemia in 31.3% of 22q11.2 deletion carriers. No relationship between plasma glutamate, glutamine, or proline and presence of psychosis, but plasma glutamate concentration positively correlated with antipsychotic dosage. Plasma glutamate higher in the lower IQ group
^1^H-MRS	Da Silva Alves et al. 2011 [72]	22q11.2 deletion	Carriers: 17 Healthy controls: 23	Mean 22q11.2 deletion with SCZ: 29.25 years (SD: 8.24) Mean 22q11.2 deletion without SCZ: 28.50 years (SD: 8.47) Mean healthy controls: 31.22 years (SD: 9.51)	Hippocampal glutamate and Glx higher in 22q11.2 deletion carriers with SCZ than those without SCZ. Hippocampal Glx higher in 22q11.2 deletion carriers with SCZ relative to healthy controls. No significant difference in the DLPFC
	Mancini et al. 2023 [73]	22q11.2 deletion	Carriers: 60 Healthy controls: 45	Mean carriers: 18.1 years (SD: 6.6) Mean controls: 16.6 years (SD: 5.1)	Increased hippocampal and striatal Glx, and decreased hippocampal GABA in 22q11.2 deletion carriers compared to controls. Deletion carriers with psychosis had higher hippocampal Glx than carriers without psychosis, and Glx increased with age only in the psychosis group. Hippocampal atrophy and BOLD variability predicted hippocampal Glx in carriers
	Mori et al. 2011 [74]	22q11.2 deletion	Carriers: 4 Healthy controls: 20	Range both groups: 2–6 years	Decreased GABA concentration in 3/4 patients
	Rice et al. 2016 [75]	Prader-Willi	Carriers: 15 Healthy controls: 15	Mean Prader-Willi with behaviour problems: 20.8 years (range: 17–30) Mean Prader-Willi without behavioural problems: 21.2 years (range: 19–29) Mean healthy controls: 21.2 years (range: 19–29)	Parietal-occipital GABA concentration was reduced in Prader-Willi syndrome participants with emotional and behavioural problems relative to Prader-Willi syndrome participants without these problems and healthy controls. Negative correlation between GABA levels and total behavioural problems
	Rogdaki et al. 2019 [76]	22q11.2 deletion	Carriers: 22 Healthy controls: 30	Mean carriers: 28.61 years (SD: 10.6) Mean controls: 27.63 years (SD: 6.02)	No group differences in Glx in the ACC, striatum, or thalamus. No association between Glx and full-scale IQ or Comprehensive Assessment of At-Risk Mental State scores
	Van Hooijdonk et al. 2022 [77]	22q11.2 deletion	Carriers: 17 Healthy controls: 20	Mean 22q11.2 deletion MRS: 34.17 years (SD: 11.41) Mean controls MRS: 30.70 years (SD: 8.20)	Glutamate, glutamine, and Glx concentrations in 22q11.2 deletion carriers were not correlated with D2/3 receptor availability. In controls, correlations between right rostral ACC volume and ACC Glu, left caudal ACC volume and ACC glutamine, and right caudal ACC volume and ACC Glx did not survive Bonferroni correction. No correlations in the carrier group
	Vingerhoets et al. 2020 [78]	22q11.2 deletion	Carriers: 17 Healthy controls: 20 (same data set as Van Hooijdonk et al.. 2022)	Mean 22q11.2 deletion: 34.17 years (SD: 11.41) Mean controls: 30.70 years (SD: 8.20)	No group-wise differences in metabolite concentrations post-placebo, with a trend towards increased ACC glutamate. Inverse correlations between ACC Glu and visual and verbal memory post-placebo, and ACC GABA and attention did not survive Bonferroni correction
*Neurophysiology*					
[^11^C]-flumazenil PET	Asahina et al. 2008 [79]	Angelman	Angelman deletion: 5 Angelman non-deletion: 2 Healthy controls: 4	Mean carriers: 17.14 years (range: 6–30) Mean healthy controls: unknown (range 22–29 years)	FMZ binding potential increased in Angelman syndrome relative to controls. Most significant elevation found in cerebral cortex and cerebellum of Angelman deletion carriers
	Holopainen et al. 2001[80]	Angelman	Angelman deletion: 3 Angelman non-deletion: 1	Mean: 7.5 years (range: 2–19)	FMZ binding potential in frontal, parietal, hippocampal, and cerebellar regions of Angelman deletion carriers was lower than the Angelman non-deletion carrier
	Lucignani et al. 2004 [81]	Prader-Willi	Carriers: 6 Healthy controls: 9	Mean carriers: 24.6 years (SD: 5.3) Mean controls: 25.9 years (SD: 3.5)	FMZ binding potential reduced in cingulate, insula, frontal, and temporal cortices of Prader-Willi syndrome participants relative to controls. Only reductions in the cingulate survived Bonferroni correction
[^123^I]-iomazenil SPECT	Mori et al. 2011 [74]	22q11.2 deletion	Carriers: 4 Healthy controls: 20	Range both groups: 2–6 years	Patients with structural brain malformations showed decreased IMZ accumulation
EEG	Donnelly et al. 2022 [82]	22q11.2 deletion	Carriers: 28 Unaffected siblings: 17	Mean carriers: 14.6 years (SD: 3.4) Mean controls: 13.7 years (SD: 3.4)	Increased low frequency power and reduced relative sigma power in 22q11.2 deletion carriers relative to controls across sleep stages. Increased spindle and slow wave amplitude mediated genotype effects on anxiety and ADHD symptoms
	Egawa et al. 2021a [83]	Angelman	Carriers: 8 Healthy controls: 11	Mean carriers: 11.6 years (SE: 2.5) Mean controls: 10.5 years (SE: 1.7)	First cortical peak latency and interval times prolonged but subcortical components unaffected in Angelman deletion carriers compared to controls
	Frohlich, Miller et al. 2019 [84]	Angelman	Angelman deletion: 37 Angelman non-deletion: 30 Healthy controls: 48	Mean Angelman deletion: 4.6 years (SD: 3.0) Mean Angelman non-deletion: 7.3 years (SD: 3.3) Mean controls: 8.8 years (SD: 5.0)	Increased theta and delta power and decreased beta power in Angelman deletion carriers compared to Angelman non-deletion carriers. Spectral power higher in Angelman syndrome compared to controls across all frequencies, with the largest difference in the delta range. EEG power higher in younger subjects
	Frohlich, Reiter et al. 2019 [85]	15q11-13 duplication	15q11-13 duplication (maternal): 27 15q11-13 duplication (paternal): 2 Child healthy controls: 14 Adult healthy controls: 12	Mean 15q11-13 interstitial duplication maternal: 80.0 months (SD: 42.7) Mean 15q11-13 isodicentric duplication maternal: 56.9 months (SD: 37.0) Mean child controls: 55 months (SD: 28.5) Adult controls: unknown	Increased peak beta power in 15q11-13 duplication carriers relative to controls. The EEG pattern in 15q11-13 duplication carriers resembled that of GABA-modulating drug midazolam in healthy adults
	Frohlich et al. 2016 [86]	15q11-13 duplication	Study 1 – carriers: 11 Study 1 – autistic controls: 10 Study 1 – healthy controls: 10 Study 2 – carriers: 27	Median carriers (study 1): 54 months (range: 16–144) Median autistic controls (study 1): unknown (range: 29–59.6 months) Median healthy controls (study 1): unknown (range: 26.6–98.7) Median carriers (study 2): 81.2 months (range: 16–384)	Increased beta power compared to both control groups in study 1, and lower delta power. Higher spontaneous gamma power in 15q11-13 duplication carriers relative to autistic controls. Autistic controls lay between duplication carriers and controls on several measures
	Larsen et al. 2018 [87]	22q11.2 deletion	Carriers: 18 Healthy controls: 27	Mean carriers: 15.39 years (SD: 2.45) Mean controls: 15.96 years (SD: 2.71)	Reductions in gamma power and inter-trial phase coherence in 22q11.2 deletion carriers. Synchronisation of gamma activity and the auditory stimulus was negatively correlated with negative symptoms of schizophrenia. The auditory steady-state response was attenuated in non-psychotic 22q11.2 deletion carriers
	Mancini et al. 2022 [88]	22q11.2 deletion	Carriers: 63 Healthy controls: 62	Mean carriers: 17.2 years (SD: 7) Mean controls: 17.3 years (SD: 6.1)	22q11.2 deletion carriers has a decreased gamma and theta power response to a visual stimulation task compared to controls. Reduced gamma band responses in 22q11.2 carriers with attenuated psychotic symptoms compared to 22q11.2 carriers without psychosis did not survive false discovery rate correction
	Mancini et al. 2022 [89]	22q11.2 deletion	Carriers: 58 Healthy controls: 58	Mean carriers: 17.6 years (SD: 6.9) Mean controls: 17.7 years (SD: 6.2)	Reduced gamma and theta band response and inter-trial phase coherence in 22q11.2 deletion carriers relative to controls. Decreases were exacerbated in 22q11.2 deletion carriers with psychosis compared to those without. Negative correlation between averaged gamma power in frontal-central electrode and hallucination subscale of Structural Interview of Prodromal Symptoms. Gamma power increased from childhood to adulthood in controls but not in deletion carriers
	Saravanapian et al. 2020 [90]	15q11-13 duplication	Study 1: 41 Study 2: 36 Study 3: 10 Study 4: 8	Range (study 1): 9–189 months	Study 1: No difference in beta band power or frequency between duplication types (interstitial vs isodicentric), with similarly increased power as previous studies. Study 2: Behavioural and cognitive measures did not predict beta power and peak frequency. Beta peak frequency was predicted by daily life skills. Study 3 & 4: good stability over time of the beta power and beta peak frequency findings, and good reproducibility between research and clinical EEG
	Saravanapian et al. 2021[91]	15q11-13 duplication	Carriers: 15 Healthy controls: 12	Mean carriers: 5.69 years (range: 9 months-13 years) Mean controls: 5.78 years (range: 7 months-14 years)	Increased beta oscillations identified in all carriers, varying with sleep stage. Beta power higher in frontal, central, and occipital regions in 15q11-13 duplication children compared to controls. 15q11-13 duplication children had reduced spindle density
MEG	Dima et al. 2020 [92]	Varied	Carriers: 42 Healthy controls: 42	Mean carriers: 38.5 years (SD: 12.5) Mean controls: 33.3 years (SD: 9.6)	Decreased oscillatory connectivity between posterior, parietal, and temporal nodes in the CNV group compared to the control group. 22q11.2 deletion carriers (N = 14) had right-hemisphere hyperconnectivity relative to controls
	Doherty et al. 2024 [93]	22q11.2 deletion	Carriers: 34 Healthy controls: 25	Mean carriers: 13.5 years (SD: 1.9) Mean controls: 14.4 years (SD: 1.8)	Decreased oscillatory activity and connectivity in the beta and gamma band in the posterior lobe of children with 22q11.2 deletion compared to controls, and increased gamma band activity in the frontal lobe. Severity of social communicative differences positively correlated with frontal gamma activity and negatively correlated with alpha and theta band connectivity. Positive correlation between alpha band activity and IQ
	Egawa et al. 2008 [94]	Angelman	Angelman deletion: 11 Angelman non-deletion: 2 Epileptic controls: 6 Healthy controls: 11	Median Angelman deletion: 10 years (range: 5–28) Median Angelman non-deletion: NA (range: 14–28 years) Median epileptic controls: 11.5 years (range: 6–24) Median healthy controls: 10 years (range: unknown)	Abnormal primary somatosensory evoked fields in all Angelman deletion carriers. Delayed and prolonged peak latency of N1m component, with stronger equivalent current dipoles (ECD). Angelman non-deletion carriers showed a similar somatosensory evoked fields pattern to controls. No correlation between ECD strength or N1m peak latency and clinical symptoms
	Egawa et al. 2021b [95]	Angelman; Prader-Willi	Angelman deletion: 11 Angelman non-deletion: 3 Prader-Willi deletion: 8 Prader-Willi non-deletion: 2 Epileptic controls: 9 Healthy controls: 11	Median Angelman deletion: 10 years (range: 5–28) Median Angelman non-deletion: 14 years (range: 9–24) Median Prader-Willi deletion: 15.5 years (range: 10–41) Median Prader-Willi non-deletion: 20 years (range: 18–24) Median epileptic controls: 11 years (range: 6–24) Median healthy controls: 10 years (range: unknown)	Delayed N1m peak latency in Angelman deletion carriers relative to all other groups, and higher N1m strength relative to controls. Normal somatosensory evoked fields in Prader-Willi syndrome. Results were unrelated to clonazepam (diazepam) prescription
iPSC and organoid models	Avazzadeh et al. 2019 [96]	2p16.3 deletion	Carriers: 3 (6 iPSC lines) Healthy controls: 5 (7 iPSC lines)	Unknown	Increased frequency, duration, and amplitude of calcium transients in 2p16.3 deletion carrier-derived cortical pyramidal neurons. Finding validated by transcriptional analysis
	Avazzadeh et al. 2021 [97]	2p16.3 deletion	Carriers: 3 (5 iPSC lines) Healthy controls: 5 (6 iPSC lines)	Unknown	Increased iPSC-derived cortical pyramidal neuron excitability in 2p16.3 deletion carriers relative to control neurons: increased action potential amplitude and faster depolarisation. Upregulation of GRIN1, GRIN3B, and glutamate transporter activity
	Fink et al. 2017 [98]	Angelman	Angelman: 3 Non-affected relatives: 4	Unknown	iPSC-derived Angelman syndrome neurons a depolarised resting membrane potential and reduced action potential amplitude, increased full-width at half maximum and reduced spike threshold relative to control neurons. Excitatory synaptic activity was similar early in development but diverged as cells matured
	Fink et al. 2021 [99]	15q11-13 duplication; Angelman	15q11-13 duplication (maternal): 4 15q11-13 duplication (paternal): 1 Angelman: 3 Healthy controls: 6	Unknown	iPSC-derived 15q11-13 duplication neurons showed hyperexcitability, characterised by increased synaptic event frequency and amplitude, increased frequency of spontaneous action potential firing, and decreased inhibitory synaptic transmission
	Hussein et al. 2023 [100]	Varied	Autistic carriers: 4 iPSC lines Non-affected relatives: 4 iPSC lines	Range carriers: 2–22 years Range controls: 39–50 years	Increased rate and amplitude of excitatory postsynaptic currents in 7q11.23 duplication carrier cell line relative to controls, alongside increased sodium and potassium currents, indicative of hyperexcitability. A higher ratio of excitatory to inhibitory cells in the patient-derived neurons across genotypes
	Khan et al. 2020 [101]	22q11.2 deletion	Carriers: 15 Healthy controls: 15	Mean carriers: 22.8 years (SD: 2.4) Mean controls: 23.8 years (SD: 4.0)	22q11.2 deletion carrier-derived neurons had an increased likelihood to spontaneously fire action potentials and a higher resting membrane potential. Enrichment of genes related to neuronal excitability, predominantly related to calcium signalling. Decreased amplitude of Ca^2+^ rise following induced depolarisation was restored by administration of antipsychotics
	Khattak et al. 2015 [102]	7q11.23 deletion	Carriers: 3 Healthy controls: 3	Unknown	7q11.23 deletion carrier-derived neurons exhibited deficits in action potential repolarisation and a smaller action potential amplitude, alongside a decrease in voltage-gated potassium currents. Upregulation of GRIK1 and downregulation of GABRA3
	Meganathan et al. 2021 [103]	15q11-13 duplication	Carriers: 2 Healthy controls: 2	Unknown	15q11-13 duplication carrier-derived neurons exhibited a larger number of action potentials and a less depolarised action potential threshold. Additional deficits in neural development: differentiation, maturation, and migration
	Nehme et al. 2022 [104]	22q11.2 deletion	Carriers: 19 Healthy controls: 29	Unknown	Decreased spiking rate in 22q11.2 deletion-carrier derived neurons and an overall reduction in network activity relative to control-derived neurons. Transcription and protein changes of genes/proteins related to ASD and SCZ risk, including NRXN1
	Pak et al. 2021 [105]	2p16.3 deletion	Carriers: 3 (5 iPSC lines) Healthy controls: 3 (6 iPSC lines)	Range carriers: 35–51 years Range controls: 39–47 years	No alterations in neuronal morphology, synapse number, neuronal excitability, resting membrane potential, or action potential generation in 2p16.3 deletion carrier-derived neurons compared to controls. Decreased frequency of miniature excitatory postsynaptic currents, with no effect on amplitude. Decreased amplitude of AMPAR-mediated excitatory postsynaptic current amplitude and neurotransmitter release probability
	Pak et al. 2015 [106]	2p16.3 deletion	Engineered, numbers unknown	Unknown	Engineered 2p16.3 deletion neurons displayed a decrease in miniature excitatory postsynaptic current frequency compared to control neurons, without a change in amplitude. No decrease in synapse number. Decreased AMPAR-mediated excitatory postsynaptic current amplitude relative to controls, and decreased neurotransmitter release probability
	Parnell et al. 2023 [107]	16p11.2 duplication	Carriers: 3 Engineered, numbers unknown	Unknown	Duplication neuron networks exhibited reduced mean firing rate, reduced synchrony, and reduced burst frequency. Transcriptomics revealed enrichment of genes associated with de novo risk variants in SCZ and ASD. Shared gene ontology between engineered and patient-derived cell lines, including enrichment of genes related to the glutamatergic synapse
	Sebastian et al. 2023 [108]	2p16.3 deletion	Organoids – carriers: 2 Organoids – controls: 2 iPSCs – carriers: 4 iPSCs – controls: 4	Unknown	2P16.3 deletion carrier-derived organoids showed a decrease in frequency of spontaneous Ca^2+^ transients, without a change in amplitude, and an overall decrease in synchronous firing rate. Differential expression of GRIN2B and failure to potentiate glycine-induced activity compared to control organoids suggests NMDA receptor disruption
	Tai et al. 2022 [109]	16p11.2 deletion; 16p11.2 duplication	Neural stem cells – 16p11.2 deletion: 7 Neural stem cells – 16p11.2 duplication: 8 Neural stem cells – WT: 12 Induced neurons – 16p11.2 deletion: 7 Induced neurons – 16p11.2 duplication: 6 Induced neurons – WT: 6 Organoids – 8 (type unknown)	Unknown	Carrier neurons (deletions and duplications) displayed reduced firing rate, synchrony, and oscillation relative to control neurons. In cerebral organoids, 16pDel exhibited more inhibitory neurons and 16pDup exhibited more excitatory neurons relative to controls
	Zhao et al. 2015 [110]	22q11.2 deletion	Carriers: 4 SCZ controls: 2 Healthy controls: 6	Range carriers: 25–41 years Range SCZ controls: 25–31 years Range healthy controls: 27–58 years	Six downregulated miRNAs in the 22q11.2 deletion carrier-derived neurons, all common to SCZ. 4/6 map to the deleted region. Genes involved in neurotransmitter function, synaptogenesis, and neuronal differentiation suggested to be affected by disruption in these miRNAs
Binocular rivalry	Choi et al. 2023 [111]	16p11.2 deletion	Carriers: 19 Healthy controls: 26	Mean carriers: 13.89 years (SD: 3.34) Mean controls: 15.69 years (SD: 6.0)	Fewer perceptual transitions and slower transition rate in 16p deletion carriers relative to neurotypical controls. Results held in autistic subgroup alone, and rivalry rate was non-significantly slower in autistic 16p11.2 deletion carriers compared to non-autistic carriers
*Neuromodulation*					
Pharmacological	Bird et al. 2021 [112]	Angelman	Carriers placebo: 26 Carriers gaboxadol (1/day): 27 Carriers gaboxadol (2/day): 25	Mean: 22.6 years (SD: 6.95)	Gaboxadol administration associated with improvement in clinical and parental global impression relative to placebo, driven by improvements in sleep
	Keary et al. 2023 [113]	Angelman	Carriers placebo: 50 Carriers gaboxadol: 47	Mean placebo: 8.1 years (SD: 2.53) Mean gaboxadol: 8.3 years (SD: 2.62)	No significant difference between gaboxadol and placebo groups of children with Angelman syndrome on any clinical measure, and no between-treatment differences in improvement from baseline
	Vingerhoets et al. 2020 [78]	22q11.2 deletion	Carriers: 17 Healthy controls: 20	Mean 22q11.2 deletion: 34.17 years (SD: 11.41) Mean controls: 30.70 years (SD: 8.20)	No group-wise differences in metabolite concentrations post-placebo, with a trend towards increased ACC glutamate. Non-significant trend towards reduced ACC Glu and significantly reduced GABA post-riluzole administration
TMS	Civardi et al. 2004 [114]	Prader-Willi	Carriers: 21 Healthy controls: 11	Mean carriers: 24.6 years (SD: 6.2) Mean healthy controls: unknown	Higher relaxed motor threshold and reduced intracortical facilitation in Prader-Willi syndrome relative to controls. Prader-Willi deletion subgroup had weaker intracortical inhibition

Where the number of participants included in each analysis differs, N refers to the total number of participants analysed in any outcome. Age statistic as reported in paper or supplementary material

*SD* standard deviation, *SE* standard error

The quality of included studies was predominantly fair (50.9%, 27 studies), followed by good (35.8%, 19 studies). A minority of studies were of poor quality (13.2%, 7 studies). Studies were judged as poor largely because of inadequate reporting of sample characteristics or inclusion criteria, and unreported or absent adjustment for confounding factors.

### Neurogenomics

In CNV carriers, neurogenomic approaches shed a light on how the genetic disorder may affect gene expression, transcription, and final protein levels. This is the first stage in a cascade of biological processes, and therefore is the earliest opportunity where a genetic abnormality might begin to affect neurobiological mechanisms. Included neurogenomics studies were limited to expression of glutamate and GABA receptor genes (listed in Table 3), as the full extent of excitatory and inhibitory synapse-related proteins is difficult to define.

**Table 3:** Glutamate and GABA receptor genes

Primary neurotransmitter	Receptor type	Receptor group	Gene(s)
Glutamate	Ionotropic	AMPA	*GRIA1*
			*GRIA2*
			*GRIA3*
			*GRIA4*
		Kainate	*GRIK1*
			*GRIK2*
			*GRIK3*
			*GRIK4*
			*GRIK5*
		NMDA	*GRIN1*
			*GRIN2A*
			*GRIN2B*
			*GRIN2C*
			*GRIN2D*
			*GRIN3A*
			*GRIN3B*
	Metabotropic	1	*GRM1*
			*GRM5*
		2	*GRM2*
			*GRM3*
		3	*GRM4*
			*GRM6*
			*GRM7*
			*GRM8*
GABA	Ionotropic (type A)	Alpha	*GABRA1*
			*GABRA2*
			*GABRA3*
			*GABRA4*
			*GABRA5*
			*GABRA6*
		Beta	*GABRB1*
			*GABRB2*
			*GABRB3*
		Delta	*GABRD*
		Epilson	*GABRE*
		Gamma	*GABRG1*
			*GABRG2*
			*GABRG3*
		Pi	*GABRP*
		Theta	*GABRQ*
		Rho	*GABRR1*
			*GABRR2*
			*GABRR3*
	Metabotropic (type B)	B	*GABBR1*
			*GABBR2*

#### Gene expression and transcriptomics

In two post-mortem studies of Angelman syndrome samples, Roden et al. [65] and Samaco et al. [66] both observed alterations of the GABAa receptor. Samaco et al. found reduced *GABRB3* expression in Angelman brain samples relative to controls. Roden, who measured *GABRB3* and *GABRB2*, found the ratio of cortical β3 (*GABRB3*) to β2 (*GABRB2*) GABAa subunit expression was reduced in Angelman syndrome compared to controls, driven by increased *GABRB2* rather than reduced *GABRB3*. Looking at the α subunits, Roden et al. found the ratio of α5 to α1 was also reduced in Angelman samples, driven by reduced *GABRA5* expression and unchanged *GABRA1*.

Reduced *GABRB3* expression has also been demonstrated in post-mortem Prader-Willi syndrome samples relative to controls [63, 64]. Alongside reduced *GABRB3* expression, Bittel et al. [63] showed increased expression of *GABRA4* and *GABRG2*, and decreased expression of *GABRA2*, *GABRA5*, and *GABRD*. Hogart et al. [64] also included two 15q11-13 duplication participants, in whom opposing patterns of *GABRB3* expression were observed: one increased and one decreased. This variability in 15q11-13 duplication was also seen in Scoles et al., who did not observe a significant difference in *GABRB3* expression between carriers and controls due to higher variability in the 15q11-13 duplication group: 3 samples had lower levels than controls and 5 had higher levels [67].

Only one study included subjects with 9p24 deletion syndrome, a very rare CNV associated with SCZ. Within one family of 21 members, 9 of whom carried a 9p24 deletion and 12 of whom did not, they found elevated expression of glutamate receptor genes – *GRIA1*, *GRIN2A*, and *GRM8* – and in particular, increased *GRIK4* expression in 5 deletion subjects with psychosis compared to 4 deletion subjects without psychosis [53].

### Neurochemistry

Neurochemical studies can directly or indirectly measure the concentrations of glutamate and GABA, and in doing so reveal both potential imbalances in excitation and inhibition, and how these concentrations may relate to symptoms of NDDs.

#### Blood and plasma glutamate and γ-aminobutyric acid (GABA)

Glutamate and GABA are preferentially removed from the brain into the blood, with little transport in the opposing direction [115, 116]. Increased brain levels of both neurotransmitters are therefore thought to contribute to elevated blood and plasma concentrations. Four studies of peripheral glutamate and GABA concentrations were retrieved: one in 15q11-13 duplication syndrome, two in Angelman and Prader-Willi syndromes, and one in 22q11.2 deletion syndrome.

Borgatti et al. [68] evaluated plasma GABA and diazepam binding inhibitor (DBI) in children with 15q11-13 duplication, mild to profound ID, and a diagnosis of ASD or pervasive developmental disorder, and age-matched healthy controls. They found no difference in concentrations of either GABA or DBI between carriers and controls. The same research group later conducted a similar study in children and young adults (age range 2–29 years) with Angelman syndrome, all of whom with a history of epileptic seizures [69]. Angelman syndrome subjects had significantly higher plasma GABA than non-epileptic controls, but significantly lower GABA than epileptic controls. Angelman syndrome participants also had higher DBI than non-epileptic controls, with no difference compared to epileptic controls. There was no difference between deletion and non-deletion Angelman subtypes. Ebert et al. [70] also evaluated plasma GABA in children and adolescents with Angelman syndrome, alongside participants with Prader-Willi syndrome, and matched controls. Angelman and Prader-Willi syndrome participants had 2–3 times higher plasma GABA concentrations than obese or intellectually disabled controls, with no differences between Angelman and Prader-Willi syndrome, between deletion and non-deletion participants, or between control groups.

Finally, Evers et al. [71] measured glutamate, glutamine, and proline in a large cohort of adults with 22q11.2 deletion syndrome, which they compared to a reference range. Only one subject had a glutamate concentration outside the reference range, and there was no association between metabolite concentrations and presence of psychosis or depression. However, glutamate concentration was positively correlated with antipsychotic dosage and was elevated in participants with relatively lower IQ. 31.3% of participants had elevated levels of the glutamate precursor proline (hyperprolinemia).

#### Proton magnetic resonance spectroscopy (^1^H-MRS)

^1^H-MRS is a magnetic resonance imaging (MRI) technique that allows quantification of metabolites in the brain. Most relevant for the study of E/I balance, both glutamate and GABA concentrations can be measured, as well as glutamine (either alone or in combination with glutamate, known as Glx). ^1^H-MRS studies of glutamate, Glx, and/or GABA in CNV populations were retrieved in 22q11.2 deletion syndrome and Prader-Willi syndrome.

Rice et al. [75] conducted the only known ^1^H-MRS study of glutamate and GABA concentrations in Prader-Willi syndrome. Consistent with plasma and post-mortem gene expression measures in Prader-Willi syndrome, they found disruption of the GABA system. Alterations were limited to reduced GABA concentration in the parieto-occipital region of Prader-Willi syndrome participants with behavioural problems compared to both typically-developing controls and Prader-Willi participants without considerable behavioural problems. GABA levels were significantly negatively correlated with total developmental behaviour checklist scores, including depressive and social relating subscales.

The evidence body of ^1^H-MRS in 22q11.2 deletion syndrome is larger, but still limited to six studies with considerable methodological variability. Da Silva Alves et al. found increased hippocampal glutamate in twelve 22q11.2 deletion carriers with schizophrenia compared to ten without schizophrenia [72]. This result was validated by Mancini et al., who found higher hippocampal and superior temporal cortex Glx, and reduced hippocampal GABA compared to healthy controls [73]. Hippocampal Glx was additionally elevated in deletion carriers with psychosis. In a considerably smaller sample, three out of four deletion carriers in Mori's study demonstrated reduced GABA concentrations relative to age-matched healthy control children [74]. In contrast, studies by Rogdaki et al. [76] and Vingerhoets et al. [78] did not find any significant differences in glutamate or GABA concentrations in the ACC, striatum, or thalamus of adults with 22q11.2 deletion compared to matched healthy controls. However, Vingerhoets et al. did find non-significant negative correlations between ACC glutamate and memory, and ACC GABA and attention. Further analysis of the Vingerhoets et al. data set by van Hooijdonk et al. did not reveal correlations between glutamate, glutamine, or Glx concentrations and dopamine receptor availability assessed by Positron Emission Tomography (PET) [77].

### Neurophysiology

Neurophysiology integrates several disciplines to study the function of the nervous system, and allows the outcomes of neurogenomic and neurochemical abnormalities to be investigated. Many of these techniques focus on the excitatory and inhibitory synapse, through the analysis of neurotransmitter receptor function, synaptic transmission, and electrical excitability.

#### Positron emission tomography (PET) and single photon emission computerised tomography (SPECT)

Both PET and SPECT use radioactive tracers designed to bind to elements of a biological system, in order to visualise its function or distribution in vivo. The affinity of the ligand to its receptor, multiplied by the maximum density of the receptor, gives the binding potential: the unit of PET and SPECT quantification. Available PET and SPECT radiotracers optimised for the investigation of the glutamate and GABA systems have been reviewed by Majo et al. (glutamate) and Andersson et al. (GABA) [117, 118]. Three PET studies of Angelman syndrome and Prader-Willi syndrome, and one further SPECT study of 22q11.2 deletion syndrome were analysed.

The three studies of Angelman and Prader-Willi syndrome used [^11^C]-flumazenil (FMZ) PET, a radioligand of the benzodiazepine binding site of GABAa receptors. All are small-scale studies, with a total of only twenty CNV carriers across all three. Two studies in Angelman syndrome studies found opposite patterns of FMZ binding potential. Asahina et al. showed greater FMZ binding potential in Angelman syndrome participants than controls, with a larger elevation in Angelman syndrome deletion carriers [79]. In contrast, Holopainen et al. found lower FMZ binding potential in Angelman syndrome deletion carriers compared to a single non-deletion Angelman syndrome participant [80]. However, neither study had a sufficient sample size to make definitive conclusions about the effect of the deletion. In a slightly larger study with nine participants per group, Lucignani et al. showed reductions in FMZ binding potential across the brain of Prader-Willi syndrome carriers, with decreases in the cingulate remaining significant post-correction for multiple comparisons [81].

Mori et al. instead used [^123^I]-iomazenil (IMZ) SPECT, which also binds to the benzodiazepine site of GABAa. Two 22q11.2 deletion syndrome carriers with structural brain malformations demonstrated decreased accumulation of IMZ relative to controls [74].

#### Electroencephalography (EEG) and magnetoencephalography (MEG)

E/I balance is thought to underlie several of the neural electrical signals detectable by electroencephalography (EEG) and magnetoencephalography (MEG), including synchronous oscillatory activity and connectivity particularly in the gamma and beta bands [119]. EEG studies were conducted in Angelman syndrome, Prader-Willi syndrome, 22q11.2 deletion syndrome, and 15q11-13 duplication syndrome.

Frohlich et al. described what they termed electrophysiological biomarkers of 15q11-13 duplication and Angelman syndrome. In children with a 15q11-13 duplication, they describe a pattern of increased spontaneous beta power and lower delta power than non-syndromic autistic and neurotypical controls [86]. In a later paper, they replicated this result: 15q11-13 duplication carrying children again showed higher peak beta power than neurotypical controls [85]. The focus of this paper, however, was demonstrating that this phenotype of elevated beta power could be modelled by administration of midazolam, a GABAa positive allosteric modulator, in healthy adults. Administration of midazolam, a benzodiazepine that enhances endogenous GABA transmission, increased beta power, with a comparable peak beta frequency in central channels as 15q11-13 duplication. Saravanapian et al. extended the work characterising this biomarker. They found beta peak frequency correlated with the daily living skills subscale of the Vineland Adaptive Behaviour Scale, but not cognitive measures, and demonstrated that the biomarker could be reliably reproduced in clinical EEG set ups [90]. Saravanapian et al. also showed that abnormal EEG patterns persist in sleep: children with 15q11-13 duplication had elevated frontal, central, and occipital beta power in all sleep stages compared to controls, alongside reduced spindle density [91].

Frohlich et al. also examined the same parameters in Angelman syndrome, comparing deletion and non-deletion variants of the syndrome [84]. They found increased EEG power in the CNV groups compared to controls, peaking in the delta range, with a greater difference for the deletion subgroup. The deletion group also had elevated theta power and decreased beta power relative to the non-deletion group.

Egawa et al. conducted three EEG and MEG studies of somatosensory-evoked potentials (SEP)/ fields (SEF) in Angelman and Prader-Willi syndromes [83, 94, 95], which they hypothesise to be related to GABA disruption. In each study they demonstrated consistent disruption of the SEF in Angelman deletion carriers, taking the form of delayed and strengthened cortical peak latency and desynchrony, relative to controls. However, this pattern was not seen in Prader-Willi syndrome, both in deletion and non-deletion carriers [95].

In sleeping children and adolescents with 22q11.2 deletion syndrome, Donnelly et al. [82] demonstrated increased EEG power at low frequencies and reduced sigma power relative to their unaffected siblings across sleep stages, as well as aberrant sleep spindles (rhythmic sigma waves occurring during non-rapid eye movement sleep). Slow wave and spindle amplitude mediated genotype effects on anxiety and ADHD symptoms. Also in 22q11.2 deletion syndrome, two relatively large-sample studies [87, 89] investigated auditory steady-state response, abnormalities of which suggest impairments in neural oscillations. Both studies demonstrated reduced gamma power and inter-trial phase coherence in CNV carriers compared to controls, and also revealed correlations between gamma activity and SCZ symptoms. Mancini et al. [89] additionally demonstrated a diverging developmental trajectory in children with 22q11.2 deletion, in whom unlike control children, gamma power did not increase with age. Evidence of abnormalities in neural oscillations in 22q11.2 deletion is strengthened by a reduction in gamma power response to visual stimuli compared to healthy controls, with a trend towards greater reduction in 22q11.2 deletion carriers with attenuated psychotic symptoms compared towards those without [88]. A similar pattern was identified using MEG, which showed decreased oscillatory connectivity in the beta and gamma band in posterior regions in children with 22q11.2 deletion compared to healthy controls, but increased gamma band activity in the frontal lobe [93]. Frontal gamma band activity was positively associated with social communicative difficulties.

In a varied adult CNV sample, the largest subgroup of whom were 22q11.2 deletion carriers but also including a smaller number of 15q11-13 CNVs, Dima [92] showed decreased MEG oscillatory connectivity in their combined CNV group compared to controls. This pattern persisted when the 22q11.2 deletion carriers were excluded.

#### Inducible pluripotent stem cells and organoids

CNV carrier-derived inducible pluripotent stem cell (iPSC) lines are becoming an increasingly popular model for NDDs, where invasive techniques can ethically be used in human tissue [120, 121]. They also provide multiple opportunities to study E/I balance, including electrophysiology, transcriptomics, and calcium imaging.

Two studies [107, 109], used CRISPR/Cas9 to induce 16p11.2 CNVs into iPSC-derived neurons, alongside control wild type cell lines and, in the case of Parnell et al. [107], 16p11.2 duplication carrier-derived cell-lines. Electrophysiology of these neuron populations showed reduced firing rate relative to control neurons, in both duplication and deletion cell lines. Synchrony of firing was also disrupted. Parnell et al. also conducted a transcriptomic analysis, finding enrichment of gene ontology terms associated with the glutamatergic synapse, alongside genes affected by de novo risk variants associated with ASD, SCZ, and BD. In their generated cerebral organoids, Tai et al. [109] reported 16p11.2 deletion organoids exhibit more inhibitory neurons and duplication organoids more excitatory neurons than the control organoids.

CRISPR/Cas9 methods were also employed by Nehme et al. [104] to induce a 22q11.2 deletion, alongside cell lines derived from 22q11.2 deletion carriers. They found that 22q11.2 deletion neurons showed a significantly lower spiking rate and reduced network activity relative to controls. Whole-cell proteomics revealed the expected reduced abundance of proteins coded within the deleted region, but also alterations of proteins associated with the presynaptic terminal. In contrast, Khan et al. reported 22q11.2 deletion carrier-derived iPSC neurons demonstrate an increased likelihood to fire action potentials and a higher resting membrane potential, both of which suggest hyperexcitability [101]. This was supported by enrichment of genes related to neuronal excitability, and a defect of calcium signalling found to be related to the alteration in resting membrane potential. A further 22q11.2 deletion iPSC study by Zhao et al. [110] focused on micro (mi) RNA expression, non-coding RNA involved in regulating gene expression. They reported downregulation of 6 miRNAs in the 22q11.2 deletion carrier-derived neurons, all of which were also downregulated in their non-CNV SCZ patient-derived cell lines. The predicted targets of these miRNAs included glutamate receptor subunits *GRIA1*, *GRIN1*, and *GRIK3*.

In five studies between 2015 and 2023, Avazzadeh et al. [96, 97], Pak et al. [105, 106], and Sebastian et al. [108] used a combination of iPSC electrophysiology and calcium imaging to study the 2p16.3 deletion. A consistent finding was a lack of alteration in neural morphology between 2p16.3 deletion cell lines and controls. However, the outcome of assessments of E/I alterations in these cell lines were less consistent. Where in their 2021 paper, Avazzadeh et al. found no difference in spontaneous excitatory post-synaptic currents (EPSC) [97], Pak et al. found a significant decrease in mini EPSC frequency and AMPA receptor-mediated EPSC amplitude. Pak et al. also found decreased neurotransmitter release probability [105, 106]. Avazzadeh et al. did however provide evidence of increased excitability in 2p16.3 deletion carrier neurons compared to controls, in the form of increased action potential amplitude and faster depolarisation. Transcription analyses by Avazzadeh et al. and Sebastian et al. found altered expression of NMDA receptor subunits, and abnormalities in the calcium transport-related genes. These findings validate further NMDA receptor and calcium transient abnormalities also reported by these studies [96, 108].

The pattern of hyperexcitability persisted in 15q11-13 duplication. In an electrophysiology study [99] hyperexcitability was exhibited in the form of increased synaptic event frequency and amplitude, and increased firing rate of spontaneous action potentials, with an expected requisite decrease in inhibitory synaptic transmission. In another paper, Meganathan reinforced the hyperexcitable phenotype [103]. Their 15q11-13 duplication carrier-derived neurons fired more action potentials than controls, with a depolarised action potential threshold. Fink et al. [98] also found diverging maturational pattens of the excitatory synapse in Angelman syndrome. Although similar to control neurons early in development, Angelman cell lines exhibited a depolarised action potential threshold and resting membrane potential, and an altered action potential shape compared to controls by 15–20 weeks in culture.

Finally, Khattak studied Williams-Beuren syndrome (7q11.23 deletion syndrome). They found deficits in action potential repolarisation and a smaller action potential amplitude, alongside defects in voltage-activated potassium currents [102]. However, there was no impairment in miEPSCs. Hussein et al. analysed iPSC lines derived from a wider ASD cohort, only one of whom was a CNV carrier: a participant with 7q11.23 duplication [100]. Cell lines derived from this participant were disproportionately excitatory, and displayed a hyperexcitable phenotype early in development, including an increased rate and amplitude of EPSCs in the duplication line relative to the unaffected relative control. Sodium and potassium currents were also increased.

### Binocular rivalry

In a binocular rivalry experimental paradigm, different images are presented simultaneously to each eye, which leads to perceptual transitions between the two images. This is thought to reflect E/I balance in the visual cortex, particularly alterations in GABA signalling. Choi et al. [111] applied this paradigm to 16p11.2 deletion carriers and age-matched neurotypical controls. They found that deletion carriers reported fewer perceptual transitions, and transition rates were non-significantly slower for 16p11.2 deletion carriers with ASD compared to those without.

### Neuromodulation

Finally, if an E/I imbalance can be demonstrated and, most importantly, reliably linked to NDD symptoms, interventions targeted at rectifying hyper- or hypoexcitability could treat these symptoms. Neuromodulatory techniques can also reveal more about the mechanisms of symptom presentation, as how an individual responds to manipulation can reflect the underlying neuronal architecture.

#### Pharmacological manipulation

Clinical trials in CNVs are hindered by low prevalence and variable phenotypic presentation. However, although typical pharmaceuticals can be effective [122], inadequate treatment of psychiatric symptoms is a considerable problem [123], as it is in idiopathic presentations. If E/I imbalance is a significant contributor to the aetiology of NDDs in CNVs, then glutamate and GABA modulating drugs may be effective at treating these symptoms, as they are for epilepsy; another common occurrence in many CNVs. Only three drug trials were retrieved that met inclusion criteria: two of gaboxadol, a GABAa receptor agonist previously suggested as a treatment for insomnia [124], and one of riluzole, a sodium-channel blocker which modulates glutamate and GABA activity [125] and is approved in the USA and EU for the treatment of amyotrophic lateral sclerosis.

Bird et al. [112] and Keary et al. [113] conducted phase 2 and phase 3 randomised double-blind clinical trials respectively of the safety and efficacy of gaboxadol in Angelman syndrome. In this phase 2 trial, gaboxadol was found to be well-tolerated in adults and adolescents with Angelman syndrome. More interestingly, an exploratory analysis showed a significant improvement in clinical global impression (CGI) scores for gaboxadol relative to placebo, largely driven by improvements in sleep [112]. However, an efficacy trial ran by Keary et al., this time in children with Angelman syndrome, did not replicate this result [113]. No significant improvement in CGI scores was observed in the gaboxadol group compared to placebo.

Vingerhoets et al. [78] studied the effect of riluzole in adults with 22q11.2 deletion syndrome. A single dose of riluzole reduced glutamate and GABA concentrations relative to placebo, only the latter being significant. A clinical trial from the same group is currently underway, investigating the effect of an 8-week riluzole intervention on E/I balance, psychosis, and cognitive symptoms in adults with 22q11.2 deletion syndrome (NL-OMON28681, https://www.onderzoekmetmensen.nl/en/trial/52172)(112).

#### Transcranial magnetic stimulation

A single study was included that employed transcranial magnetic stimulation (TMS) [114], positing that the TMS relaxed motor threshold (the stimulation intensity required to induce a motor response) reflects corticospinal excitability. Investigating participants with Prader-Willi syndrome, the authors found a significantly higher motor threshold and weaker intracortical inhibition compared to healthy controls.

## Discussion

The available literature body reflects four key aspects of excitation/inhibition balance in copy number variant syndromes: neurogenomics, neurochemistry, neurophysiology, and neuromodulation. E/I imbalance does indeed appear to be disrupted in CNV populations, across modalities. Furthermore, the evidence reinforces E/I imbalance as an important mechanism in NDD pathology.

### Excitation/inhibition imbalance, copy number variants, and neurodevelopmental disorders

Studies analysing the relationship between NDD symptoms and E/I imbalance paint a picture of increased imbalance being associated with increased symptomatic severity. Hyperexcitability, and increased glutamatergic transmission relative to GABAergic transmission, appears to be associated with more severe psychotic symptomatology. Da Silva Alves et al. [72] and Mancini et al. [73] both found Glx concentration was higher in 22q11.2 deletion carriers with SCZ compared to deletion carriers without SCZ. Afshari et al. [53] observed increased expression of kainate receptor *GRIK4* in 9p24.2 deletion carriers with psychosis compared to those without. Although Evers et al. [71] did not find plasma glutamate concentration correlated with psychotic symptoms, it did positively correlate with antipsychotic dosage, which could reflect a predisposition to increased glutamate in those 22q11.2 deletion carriers who require treatment for psychotic symptoms. This suggests consistency between CNV populations and patients with idiopathic schizophrenia, in which the degree of hyperglutamatergia has been shown to correlate with psychotic symptom severity and is mediated by antipsychotic medication [29].

Abnormalities in glutamate and GABA may also be associated with cognitive and behavioural disturbances. Evers reported increased glutamate in their low IQ subgroup [71]. Rice observed a negative correlation between parietal-occipital GABA levels and behavioural problems in Prader-Willi syndrome [75]. Vingerhoets et al. found ACC glutamate concentration to be inversely correlated with memory and ACC GABA concentration to be inversely correlated with attention [78]. Although neither of these relationships survived Bonferroni correction, these results are interesting when considered alongside those of Donnelly et al. [82], who found an altered relationship between sleep features and accuracy on a memory recall task in youth with a 22q11.2 deletion compared to controls. NMDA and AMPA receptor circuitry has a well-established role in long-term potentiation, and although the relationship between glutamate, GABA, and sleep is undoubtedly complex, memory consolidation post-sleep also appears to be dependent on NMDA and AMPA receptor-mediated signal transduction [126]. GABAa receptors too, have an important role in sleep induction and maintenance, as well as coordinating the oscillatory activity necessary for memory consolidation [126].

In waking encephalography too, does the magnitude of the abnormality in CNV populations predict NDD symptoms. In a non-psychotic 22q11.2 deletion sample, Larsen et al. [87] found aberrant oscillatory activity was related the negative symptoms subscale of the Positive and Negative Syndrome Scale (PANSS) of SCZ symptoms. In a similar paradigm, Mancini et al. demonstrated an exaggerated phenotype in 22q11.2 deletion carriers with SCZ compared to those without, which was correlated with the severity of hallucinations [89]. ASD symptoms too, were shown to relate to oscillatory abnormalities, with a positive correlation between social communicative difficulties and gamma band power [93].

### Implications for understanding neurodevelopmental disorders

Although there is a clear experimental link between E/I imbalance and both idiopathic and syndromic NDD pathology, how exactly diverse observations such as altered glutamate to GABA ratios or aberrant gamma band oscillations create or contribute to NDD symptom manifestation remains an open question. Reduced cortical inhibition, perhaps due to disrupted maturation of GABAergic circuitry in neurodevelopmental critical periods [23], leads to noisier circuitry, less efficient information processing, and altered synaptic plasticity [20, 127]. If NDDs are viewed as a spectrum of possible outcomes that may follow neurodevelopmental disruption [128], the precise nature of this disruption may differ between CNV syndromes and between individuals with and without CNVs, but lead to similar patterns of dysfunction. For example, if the *PRODH* deletion in 22q11.2 deletion syndrome may lead to hyperexcitation, and the deletion of GABA receptor genes in Angelman syndrome may lead to hypoinhibition, an increased E/I ratio is observed in both. Different neural abnormalities may therefore have a similar overall outcome, and these may be a result of genetic or environmental pressures. In this context, CNVs are simply another (albeit unusually impactful) genetic factor which could lead to E/I imbalance, in a complex profile of genetic and environmental factors that control the manifestation of NDDs in any given individual. Indeed, the subtleties of this individual profile are likely what determines differing phenotypes between and within CNVs, where some people may develop one or another NDD.

The evidence of E/I imbalance in CNVs, and its speculative relationship to symptoms, suggests that this may be a promising avenue for treatment. Glutamatergic and GABAergic treatments of neurodevelopmental disorders are still in their infancy, but over coming years are expected to rise in prominence, given the inadequacies of many conventional pharmaceutical therapies [123, 129]. Drugs that act on the glutamate and GABA systems, although predominantly prescribed as anticonvulsants, often have secondary or off-label use as antipsychotics and mood-stabilisers. For example, valproate, lamotrigine, and carbamazepine all interact with sodium channels, typically with glutamate-supressing effects, and in the case of valproate are thought to inhibit enzymes that remove GABA from the synapse [130]. There is also preliminary evidence, currently on a case-level, that glutamatergic and GABAergic pharmacy may have a positive effect in a subgroup of CNV carriers. In Vingerhoets et al.'s single-dose riluzole cohort [78], a young woman with 22q11.2 deletion syndrome reported their hallucinations disappeared. In the weeks following her participation, her hallucinations returned, and she decided to continue the study medication. At 18-month follow-up, the participant no longer reported any hallucinations or paranoid ideas, alongside improvement in cognitive domains [131]. A randomised controlled trial of riluzole as an adjunct therapy to risperidone in chronic idiopathic SCZ did not find an effect on positive symptoms of psychosis but did show a significant improvement in negative and generalised psychopathology symptoms [132]. This suggests riluzole may have potential benefit in (a subgroup of) 22q11.2 deletion syndrome carriers and perhaps idiopathic SCZ patients, and future larger-scale research could help to identify the characteristics that define this group. Clinical trials of E/I targeted drugs for psychiatric symptoms in 22q11.2 deletion syndrome are currently underway, including of riluzole (NL-OMON28681, https://www.onderzoekmetmensen.nl/en/trial/52172) [133] and fasoracetam, a metabotropic glutamate receptor agonist already suggested to be effective in adolescents with ADHD and mutations of metabotropic glutamate receptor network genes [134, 135] (NCT05290493, https://clinicaltrials.gov/study/NCT05290493). Non-invasive brain stimulation (NIBS) may also prove a valuable avenue for treating E/I imbalance on a network level. In idiopathic childhood ASD and ADHD, this approach has shown promise, lessening EEG abnormalities associated with E/I imbalance and improving cognitive performance [136]. In 2024, Latrèche et al., published the first known transcranial direct current stimulation study in 22q11.2 deletion syndrome, and demonstrated positive effects on visual working memory [137].

### Limitations

#### Limitations of evidence body

By definition, the prevalence of the syndromes under discussion in this review is very low. This makes recruitment for CNV studies challenging, compounded by the additional difficulties recruiting and testing participants with NDDs, particularly children and those with limited capacity to consent. These challenges are especially relevant for ^1^H-MRS, SPECT, and PET studies, perhaps the most direct techniques to measure human E/I imbalance in vivo, where safety contraindications (the rate of which is increased by the physical phenotype of CNVs) significantly limit recruitment, and self-select a healthier subset of the population. Recruitment and inclusion difficulties have likely contributed to the small sample sizes in the majority of studies included in this review. Within the limited samples, several studies make comparisons within the CNV group, divided by psychiatric symptoms or by CNV type. Whilst these analyses are valuable, the statistical power is reduced further by the subdivision and often insufficient. Moreover, although only CNVs common enough to recruit a study sample can be investigated with any kind of statistical rigor, rarer syndromes could also convey risk through related mechanisms, emphasising the importance of multi-centre collaboration to increase sample size. The complexity of CNV phenotypes, where an individual may have many co-occurring health conditions, additionally complicates recruitment of a matched control group. This leads to complicated designs, for example with non-syndromic epileptic or NDD controls alongside typically developing controls, further reducing the power of statistical analysis. Conspicuous in its absence, is the 22q11.2 duplication syndrome, affecting the same chromosomal region as the equivalent deletion syndrome and associated an increased (albeit to a lesser extent) risk of NDDs, including ADHD and ASD. Inclusion of the duplication syndrome in future studies could give the same dual perspective on the effect of the candidate gene dosage lent for example by the concurrent study of both 15q11-13 deletion and duplication syndromes.

Measurement of E/I balance also has several limitations. Some methods are indirect, measuring peripheral or up- and downstream markers. Others only reflect a supposed emergent property of E/I balance, such as binocular rivalry and EEG components. There is also considerable evidence of variability between brain regions, and many techniques only assess a limited region of interest. Even cellular electrophysiology methods that can directly assess neural excitation are often conducted in isolated cell populations and struggle to represent complex human development. Few studies combined measures of excitation and inhibition to give an estimate of the balance between the two, or of compensatory action as a consequence of disruption to either system.

Although a primary aim of this review was to evaluate the connection between E/I balance and NDD symptoms, only a minority of the retrieved studies conducted any symptom assessment. When symptom assessment was present, this typically concerned psychotic symptom presentation, with features of for example executive dysfunction or social functioning less frequently studied. When analysed, the correlation between E/I imbalance and symptoms was often inconsistent, even when there was a clear difference between carriers and controls on the E/I measure. On a related note, although not an inclusion criterion for study selection in this review, participant selection for many included studies was biased towards those with a NDD diagnosis. Bearing in mind the phenotypic variability in CNVs, implementing a diagnosis requirement results in a misrepresentation of the true population. It is common in CNV populations for identification of the CNV or the first NDD diagnosis (often ID) to overshadow diagnosis of additional comorbidities [138], meaning that once a CNV or ID is diagnosed, the individual will not be assessed for other conditions. As well as limiting their clinical treatment, this can result in exclusion of potential participants who have not been diagnosed but would meet diagnostic criteria. Diagnostic requirements also exclude participants with sub-threshold or prodromal symptoms, who may still provide valuable mechanistic insights. However, when such requirements are not imposed, the subgroup with above threshold NDD symptoms is often too small to conduct robust analyses. A possible alternative is implementation of a structured clinical interview prior to inclusion to identify undiagnosed pathologies, with the drawback of additional time and personnel costs.

#### Limitations of the current review

While every effort has been made to accurately represent the literature base on this topic, many related factors were outside of the scope of this review. The evidence described does not fully capture the complex interactions between excitatory and inhibitory systems. Glutamate and GABA systems do not exist in a vacuum, and in isolation from other neural processes (for example dopamine and serotonin systems) cannot fully explain the complex NDD phenotype in CNV syndromes. Many CNVs have known associations with other neural pathways, which likely interact with and complement E/I imbalance. An example of this is catechol-o-methyltransferase (COMT), an enzyme responsible for degradation dopamine and norepinephrine at the synapse [139]. The *COMT* gene lies in locus 11.2 of chromosome 22, and so is deleted in the majority of 22q11.2 deletion carriers. The dual deletion of *COMT* and *PRODH* motivates studies such as van Hooijdonk et al. [77], which investigate glutamate and dopamine irregularities in tandem. A second case is ubiquitin protein ligase E3A (*UBE3A*), found within region 15q11-13 and typically regarded as causative for the phenotypes associated with variation in its copy number [140]. UBE3A targets proteins for proteasomal degradation and has been implicated in a myriad of cellular processes, including translation, transcription, and intracellular trafficking. Perhaps mostly relevantly, UBE3A interacts with small-conductance potassium channels. Increase in small-conductance potassium channels as a result of UBE3A loss has been suggested to decrease NMDA receptor activation and increase EEG delta power [140]. The resulting picture is an intricate network of regulatory and feedback loops, downstream consequences, and varied polygenic mechanisms. Further, syndromic CNV carriers may also harbour other genetic risk factors for NDDs, including the SNPs, SNVs, and non-syndromic CNVs that make up a significant proportion of the genetic risk, many of which are related to E/I balance [141–143]. This complexity likely explains a great deal of the variability in patterns of E/I activity in the studies discussed in this review, as well as much of the substantial phenotypic variability seen in CNV populations. In this context, the study of SNVs makes for a far simpler interpretation, where the specific effects of alterations in the activity of a single gene may be investigated.

Also beyond the scope of the current review were studies of the wider tripartite excitatory and inhibitory synapses, including broader pre- and post-synaptic complex proteins and glial activity, as well as studies of epilepsy and epileptic activity, unless NDDs were also discussed. Epilepsy is common in several of the CNV populations studied, and is associated with lower IQ and cognitive impairments, influencing the NDD phenotype in CNV carriers. As epilepsy is known to reflect disruption of E/I balance, it is likely inclusion of the epilepsy evidence body could enhance understanding of hyperexcitability in these CNVs. Conversely, the breadth of E/I measurement techniques and CNVs that were eligible for inclusion limited the depth of analysis of the mechanism of any individual pathway. A great amount of high-quality work has been conducted in animal models of NDDs, many of which use an induced CNV to model disease processes. Whilst animal research does tend to support findings in human populations [144–146]; these studies were also excluded for two reasons. Firstly, like cellular models, animal models cannot accurately represent human NDD symptoms, for example hallucinations or theory of mind. Secondly, and unlike human cellular models, the model species genome is not identical to the human genome, and although there may be substantial homology, genetic abnormality will not have identical outcomes cross-species.

### Future directions

Future investigation of E/I imbalance in CNVs should employ the full range of available techniques designed for this purpose, as well as adopting new methods as they are developed. One such existing method that could complement the current evidence body is functional MRS: a technique by which dynamic concentrations of glutamate and GABA can be tracked in response to external stimuli [147]. Cognitive paradigms would be of particular use, whereby potentially altered neurochemical response to a given task between carriers and controls could be studied. This technique has demonstrated its usefulness in SCZ, BD, and major depressive disorder [148, 149]. Furthermore, given the observed regional variability in glutamate and GABA concentrations, chemical shift imaging (CSI) could be implemented to record multi-voxel spectra in slices or the whole brain. Glutamate-related PET and SPECT tracers, not used in any of the included studies, whilst invasive, could complement the existing GABAergic work and reveal corresponding glutamate patterns. Functional connectivity too, may correlate with E/I dynamics across the brain, as measured by ^1^H-MRS, emphasising the future utility of multimodal MRI in CNV studies [150–152].

Perhaps most impactful in this field, however, would be the implementation of multi-site and consortia studies to increase the statistical power and therefore generalisability of findings. Several such projects are already underway in CNV research, including the Genes to Mental Health network (G2MH, https://genes2mentalhealth.com/), studying neurobehavioural and cognitive symptoms of genetic variants associated with NDDs. Large-scale collaborations such as G2MH and psychiatric genetics consortia may elucidate the mechanisms by which genetic factors such as SNVs and CNVs convey NDD risk. Such projects can also facilitate longitudinal research, as well as widen the opportunity for multimodal and cross-sectional studies. Longitudinal studies are essential for the understanding of neurodevelopmental disorders. Cross-sectional research tracking the trajectory of neurodevelopmental disorder manifestation in 22q11.2 [153, 154] and 16p11.2 [155] copy number variation has been conducted, but within-subjects longitudinal research would provide meaningful insight into the mechanisms of neurodevelopmental pathology. Post-mortem research also has an important place in this sphere, allowing direct, invasive investigation of neurobiological processes and generation of specific evidence-based hypotheses, facilitated by the inception of brain bank projects [156].

### Conclusions

There is sufficient evidence that excitation-inhibition imbalance holds promise as a candidate mechanism of neurodevelopmental disorder pathogenesis in copy number variant syndromes. Further, this review reinforces the value of CNV studies to understand the pathogenic mechanisms of NDDs in the general population. However, at present there is still much we do not understand about the extent of excitation-inhibition imbalance in CNVs, and how this may interact with other neurobiological and environmental processes to create the highly variable phenotype we see in these populations. Nevertheless, identification of a common mechanism between these highly related disorders, and one that is reflected in the CNV syndromes that are such strong risk factors for their manifestations, may be a turning point for the treatment of neurodevelopmental disorders.

## Supplementary Information

Supplementary Material 1.

## Data Availability

No datasets were generated or analysed during the current study.

## Abbreviations

ACC: Anterior cingulate cortex
ADHD: Attention deficit hyperactivity disorder
AMPA: α-Amino-3-hydroxy-5-methyl-4-isoxazolepropionic acid
ASD: Autism spectrum disorder
BD: Bipolar disorder
COMT: Catechol-o-methyltransferase
CNV: Copy number variant
CSI: Chemical shift imaging
DBI: Diazepam binding inhibitor
DLPFC: Dorsolateral prefrontal cortex
EEG: Electroencephalography
E/I: Excitation/inhibition
EPSC: Excitatory post-synaptic current
FMZ: Flumazenil
GABA: γ-Aminobutyric acid
^1^H-MRS: Proton magnetic resonance spectroscopy
ID: Intellectual disability
IMZ: Iomazenil
iPSC: Inducible pluripotent stem cell
MEG: Magnetoencephalography
miRNA: Micro ribonucleic acid
MRI: Magnetic resonance imaging
mRNA: Messenger ribonucleic acid
NDD: Neurodevelopmental disorder
NIBS: Non-invasive brain stimulation
NMDA: N-methyl-D-aspartate
PET: Positron emission tomography
PRODH: Proline dehydrogenase
SCZ: Schizophrenia
SEF: Somatosensory-evoked field
SEP: Somatosensory-evoked potential
SNP: Single nucleotide polymorphism
SNV: Single nucleotide variant
SPECT: Single photon emission computed tomography
TMS: Transcranial magnetic stimulation
UBE3A: Ubiquitin protein ligase E3A
WT: Wild type

## References

[1] Murray R, Lewis S. Is schizophrenia a neurodevelopmental disorder? BMJ. 1987;295(6600):681–2. 3117295 10.1136/bmj.295.6600.681 PMC1247717

[2] Weinberger DR. Implications of normal brain development for the pathogenesis of schizophrenia. In: Nasrallah HA, Weinberger DR, editors. The neurology of schizophrenia. Amsterdam: Elsevier; 1986.

[3] Rapoport JL, Addington AM, Frangou S, Psych MR. The neurodevelopmental model of schizophrenia: update 2005. Mol Psychiatry. 2005;10(5):434–49. 15700048 10.1038/sj.mp.4001642

[4] Rapoport JL, Giedd JN, Gogtay N. Neurodevelopmental model of schizophrenia: update 2012. Mol Psychiatry. 2012;17(12):1228–38. 22488257 10.1038/mp.2012.23 PMC3504171

[5] Kloiber S, Rosenblat JD, Husain MI, Ortiz A, Berk M, Quevedo J, et al. Neurodevelopmental pathways in bipolar disorder. Neurosci Biobehav Rev. 2020;112:213–26. 32035092 10.1016/j.neubiorev.2020.02.005

[6] Sanches M, Keshavan MS, Brambilla P, Soares JC. Neurodevelopmental basis of bipolar disorder: a critical appraisal. Prog Neuropsychopharmacol Biol Psychiatry. 2008;32(7):1617–27. 18538910 10.1016/j.pnpbp.2008.04.017

[7] Moreno-De-Luca A, Myers SM, Challman TD, Moreno-De-Luca D, Evans DW, Ledbetter DH. Developmental brain dysfunction: revival and expansion of old concepts based on new genetic evidence. Lancet Neurol. 2013;12(4):406–14. 23518333 10.1016/S1474-4422(13)70011-5 PMC4013791

[8] Hartung CM, Lefler EK. Sex and gender in psychopathology: DSM-5 and beyond. Psychol Bull. 2019;145(4):390–409. 30640497 10.1037/bul0000183

[9] Homberg JR, Kyzar EJ, Stewart AM, Nguyen M, Poudel MK, Echevarria DJ, et al. Improving treatment of neurodevelopmental disorders: recommendations based on preclinical studies. Expert Opin Drug Discov. 2016;11(1):11–25. 26558752 10.1517/17460441.2016.1115834

[10] Cardoso AR, Lopes-Marques M, Silva RM, Serrano C, Amorim A, Prata MJ, et al. Essential genetic findings in neurodevelopmental disorders. Hum Genomics. 2019;13(1):31. 31288856 10.1186/s40246-019-0216-4 PMC6617629

[11] Smeland OB, Bahrami S, Frei O, Shadrin A, O’Connell K, Savage J, et al. Genome-wide analysis reveals extensive genetic overlap between schizophrenia, bipolar disorder, and intelligence. Mol Psychiatry. 2020;25(4):844–53. 30610197 10.1038/s41380-018-0332-x PMC6609490

[12] Sonderby IE, Ching CRK, Thomopoulos SI, van der Meer D, Sun D, Villalon-Reina JE, et al. Effects of copy number variations on brain structure and risk for psychiatric illness: Large-scale studies from the ENIGMA working groups on CNVs. Hum Brain Mapp. 2022;43(1):300–28. 33615640 10.1002/hbm.25354 PMC8675420

[13] Wilfert AB, Sulovari A, Turner TN, Coe BP, Eichler EE. Recurrent de novo mutations in neurodevelopmental disorders: properties and clinical implications. Genome Med. 2017;9(1):101. 29179772 10.1186/s13073-017-0498-x PMC5704398

[14] Malhotra D, Sebat J. CNVs: harbingers of a rare variant revolution in psychiatric genetics. Cell. 2012;148(6):1223–41. 22424231 10.1016/j.cell.2012.02.039 PMC3351385

[15] Rees E, Walters JT, Georgieva L, Isles AR, Chambert KD, Richards AL, et al. Analysis of copy number variations at 15 schizophrenia-associated loci. Br J Psychiatry. 2014;204(2):108–14. 24311552 10.1192/bjp.bp.113.131052 PMC3909838

[16] Marshall CR, Howrigan DP, Merico D, Thiruvahindrapuram B, Wu W, Greer DS, et al. Contribution of copy number variants to schizophrenia from a genome-wide study of 41,321 subjects. Nat Genet. 2017;49(1):27–35. 27869829 10.1038/ng.3725 PMC5737772

[17] Rylaarsdam L, Guemez-Gamboa A. Genetic causes and modifiers of autism spectrum disorder. Front Cell Neurosci. 2019;13:385. 31481879 10.3389/fncel.2019.00385 PMC6710438

[18] Gudmundsson OO, Walters GB, Ingason A, Johansson S, Zayats T, Athanasiu L, et al. Attention-deficit hyperactivity disorder shares copy number variant risk with schizophrenia and autism spectrum disorder. Transl Psychiatry. 2019;9(1):258. 31624239 10.1038/s41398-019-0599-y PMC6797719

[19] Zarrei M, Burton CL, Engchuan W, Young EJ, Higginbotham EJ, MacDonald JR, et al. A large data resource of genomic copy number variation across neurodevelopmental disorders. NPJ Genom Med. 2019;4:26. 31602316 10.1038/s41525-019-0098-3 PMC6779875

[20] Sohal VS, Rubenstein JLR. Excitation-inhibition balance as a framework for investigating mechanisms in neuropsychiatric disorders. Mol Psychiatry. 2019;24(9):1248–57. 31089192 10.1038/s41380-019-0426-0 PMC6742424

[21] Bethlehem RAI, Seidlitz J, White SR, Vogel JW, Anderson KM, Adamson C, et al. Brain charts for the human lifespan. Nature. 2022;604(7906):525–33. 35388223 10.1038/s41586-022-04554-y PMC9021021

[22] Caballero A, Tseng KY. GABAergic function as a limiting factor for prefrontal maturation during adolescence. Trends Neurosci. 2016;39(7):441–8. 27233681 10.1016/j.tins.2016.04.010 PMC4930717

[23] Reh RK, Dias BG, Nelson CA 3rd, Kaufer D, Werker JF, Kolb B, et al. Critical period regulation across multiple timescales. Proc Natl Acad Sci U S A. 2020;117(38):23242–51. 32503914 10.1073/pnas.1820836117 PMC7519216

[24] Hensch TK. Critical period plasticity in local cortical circuits. Nat Rev Neurosci. 2005;6(11):877–88. 16261181 10.1038/nrn1787

[25] Zhang S, Larsen B, Sydnor VJ, Zeng T, An L, Yan X, et al. In vivo whole-cortex marker of excitation-inhibition ratio indexes cortical maturation and cognitive ability in youth. Proc Natl Acad Sci U S A. 2024;121(23):e2318641121. 38814872 10.1073/pnas.2318641121 PMC11161789

[26] Meredith RM. Sensitive and critical periods during neurotypical and aberrant neurodevelopment: a framework for neurodevelopmental disorders. Neurosci Biobehav Rev. 2015;50:180–8. 25496903 10.1016/j.neubiorev.2014.12.001

[27] Canitano R, Pallagrosi M. Autism spectrum disorders and schizophrenia spectrum disorders: excitation/inhibition imbalance and developmental trajectories. Front Psychiatry. 2017;8:69. 28507523 10.3389/fpsyt.2017.00069 PMC5410649

[28] Dickinson A, Jones M, Milne E. Measuring neural excitation and inhibition in autism: different approaches, different findings and different interpretations. Brain Res. 2016;1648(Pt A):277–89. 27421181 10.1016/j.brainres.2016.07.011

[29] Foss-Feig JH, Adkinson BD, Ji JL, Yang G, Srihari VH, McPartland JC, et al. Searching for cross-diagnostic convergence: neural mechanisms governing excitation and inhibition balance in schizophrenia and autism spectrum disorders. Biol Psychiatry. 2017;81(10):848–61. 28434615 10.1016/j.biopsych.2017.03.005 PMC5436134

[30] Gao R, Penzes P. Common mechanisms of excitatory and inhibitory imbalance in schizophrenia and autism spectrum disorders. Curr Mol Med. 2015;15(2):146–67. 25732149 10.2174/1566524015666150303003028 PMC4721588

[31] Hollestein V, Poelmans G, Forde NJ, Beckmann CF, Ecker C, Mann C, et al. Excitatory/inhibitory imbalance in autism: the role of glutamate and GABA gene-sets in symptoms and cortical brain structure. Transl Psychiatry. 2023;13(1):18. 36681677 10.1038/s41398-023-02317-5 PMC9867712

[32] Rubenstein JL, Merzenich MM. Model of autism: increased ratio of excitation/inhibition in key neural systems. Genes Brain Behav. 2003;2(5):255–67. 14606691 10.1034/j.1601-183x.2003.00037.x PMC6748642

[33] Merritt K, McCutcheon RA, Aleman A, Ashley S, Beck K, Block W, et al. Variability and magnitude of brain glutamate levels in schizophrenia: a meta and mega-analysis. Mol Psychiatry. 2023;28(5):2039–48. 36806762 10.1038/s41380-023-01991-7 PMC10575771

[34] Merritt K, McGuire PK, Egerton A, Aleman A, Block W, Bloemen OJN, et al. Association of age, antipsychotic medication, and symptom severity in schizophrenia with proton magnetic resonance spectroscopy brain glutamate level: a mega-analysis of individual participant-level data. JAMA Psychiat. 2021;78(6):667–81. 10.1001/jamapsychiatry.2021.0380 PMC8060889 33881460

[35] Neo WS, Foti D, Keehn B, Kelleher B. Resting-state EEG power differences in autism spectrum disorder: a systematic review and meta-analysis. Transl Psychiatry. 2023;13(1):389. 38097538 10.1038/s41398-023-02681-2 PMC10721649

[36] Mamiya PC, Arnett AB, Stein MA. Precision medicine care in ADHD: the case for neural excitation and inhibition. Brain Sci. 2021;11(1):91. 33450814 10.3390/brainsci11010091 PMC7828220

[37] Martino M, Magioncalda P. Tracing the psychopathology of bipolar disorder to the functional architecture of intrinsic brain activity and its neurotransmitter modulation: a three-dimensional model. Mol Psychiatry. 2022;27(2):793–802. 33414499 10.1038/s41380-020-00982-2

[38] Gigante AD, Bond DJ, Lafer B, Lam RW, Young LT, Yatham LN. Brain glutamate levels measured by magnetic resonance spectroscopy in patients with bipolar disorder: a meta-analysis. Bipolar Disord. 2012;14(5):478–87. 22834460 10.1111/j.1399-5618.2012.01033.x

[39] Edden RAE, Crocetti D, Zhu H, Gilbert DL, Mostofsky SH. Reduced GABA concentration in attention-deficit/hyperactivity disorder. Arch Gen Psychiatry. 2012;69(7):750–3. 22752239 10.1001/archgenpsychiatry.2011.2280 PMC3970207

[40] Puts NA, Ryan M, Oeltzschner G, Horska A, Edden RAE, Mahone EM. Reduced striatal GABA in unmedicated children with ADHD at 7T. Psychiatry Res Neuroimaging. 2020;301:111082. 32438277 10.1016/j.pscychresns.2020.111082

[41] Tseng HH, Wu CY, Chang HH, Lu TH, Chang WH, Hsu CF, et al. Posterior cingulate and medial prefrontal excitation-inhibition balance in euthymic bipolar disorder. Psychol Med. 2024;54(11):3168–76. 38825858 10.1017/S0033291724001326

[42] Cohen SM, Nadler JV. Proline-induced potentiation of glutamate transmission. Brain Res. 1997;761:271–82. 9252026 10.1016/s0006-8993(97)00352-1

[43] Flore G, Cioffi S, Bilio M, Illingworth E. Cortical development requires mesodermal expression of tbx1, a gene haploinsufficient in 22q11.2 deletion syndrome. Cerebral Cortex. 2017;27(3):2210–25. 27005988 10.1093/cercor/bhw076

[44] Poot M, Eleveld MJ, van ’t Slot R, Ploos van Amstel HK, Hochstenbach R. Recurrent copy number changes in mentally retarded children harbour genes involved in cellular localization and the glutamate receptor complex. Eur J Hum Genet. 2010;18(1):39–46. 19623214 10.1038/ejhg.2009.120 PMC2987154

[45] Pocklington AJ, Rees E, Walters JT, Han J, Kavanagh DH, Chambert KD, et al. Novel findings from CNVs implicate inhibitory and excitatory signaling complexes in Schizophrenia. Neuron. 2015;86(5):1203–14. 26050040 10.1016/j.neuron.2015.04.022 PMC4460187

[46] Cherlyn SY, Woon PS, Liu JJ, Ong WY, Tsai GC, Sim K. Genetic association studies of glutamate, GABA and related genes in schizophrenia and bipolar disorder: a decade of advance. Neurosci Biobehav Rev. 2010;34(6):958–77. 20060416 10.1016/j.neubiorev.2010.01.002

[47] Bramer WM, Giustini D, de Jonge GB, Holland L, Bekhuis T. De-duplication of database search results for systematic reviews in EndNote. J Med Libr Assoc. 2016;104(3):240–3. 27366130 10.3163/1536-5050.104.3.014 PMC4915647

[48] Ryan R, Synnot A, Prictor M, Hill S. Cochrane Consumers and Communication Group data extraction template for included studies. Melbourne: La Trobe University; 2016.

[49] Lowther C, Speevak M, Armour CM, Goh ES, Graham GE, Li C, et al. Molecular characterization of NRXN1 deletions from 19,263 clinical microarray cases identifies exons important for neurodevelopmental disease expression. Genet Med. 2017;19(1):53–61. 27195815 10.1038/gim.2016.54 PMC4980119

[50] Merla G, Brunetti-Pierri N, Micale L, Fusco C. Copy number variants at Williams-Beuren syndrome 7q11.23 region. Hum Genet. 2010;128(1):3–26. 20437059 10.1007/s00439-010-0827-2

[51] Serrano-Juarez CA, Prieto-Corona B, Rodriguez-Camacho M, Sandoval-Lira L, Villalva-Sanchez AF, Yanez-Tellez MG, et al. Neuropsychological genotype-phenotype in patients with williams syndrome with atypical deletions: a systematic review. Neuropsychol Rev. 2023;33(4):891–911. 36520254 10.1007/s11065-022-09571-2

[52] Velleman SL, Mervis CB. Children with 7q11.23 duplication syndrome: speech, language, cognitive, and behavioral characteristics and their implications for intervention. Perspect Lang Learn Educ. 2011;18(3):108–16. 22754604 10.1044/lle18.3.108 PMC3383616

[53] Afshari P, Myles-Worsley M, Cohen OS, Tiobech J, Faraone SV, Byerley W, et al. Characterization of a novel mutation in SLC1A1 associated with Schizophrenia. Mol Neuropsychiatry. 2015;1(3):125–44. 26380821 10.1159/000433599 PMC4568439

[54] Sams EI, Ng JK, Tate V, Claire Hou YC, Cao Y, Antonacci-Fulton L, et al. From karyotypes to precision genomics in 9p deletion and duplication syndromes. HGG Adv. 2022;3(1):100081. 35047865 10.1016/j.xhgg.2021.100081 PMC8756500

[55] Bird LM. Angelman syndrome: review of clinical and molecular aspects. Appl Clin Genet. 2014;7:93–104. 24876791 10.2147/TACG.S57386 PMC4036146

[56] Cassidy SB, Schwartz S, Miller JL, Driscoll DJ. Prader-Willi syndrome. Genet Med. 2012;14(1):10–26. 22237428 10.1038/gim.0b013e31822bead0

[57] Parijs I, Brison N, Vancoillie L, Baetens M, Blaumeiser B, Boulanger S, et al. Population screening for 15q11-q13 duplications: corroboration of the difference in impact between maternally and paternally inherited alleles. Eur J Hum Genet. 2024;32(1):31–6. 37029316 10.1038/s41431-023-01336-6 PMC10772068

[58] Kalsner L, Chamberlain SJ. Prader-Willi, Angelman, and 15q11-q13 Duplication Syndromes. Pediatr Clin North Am. 2015;62(3):587–606. 26022164 10.1016/j.pcl.2015.03.004 PMC4449422

[59] Vos N, Kleinendorst L, van der Laan L, van Uhm J, Jansen PR, van Eeghen AM, et al. Evaluation of 100 Dutch cases with 16p11.2 deletion and duplication syndromes; from clinical manifestations towards personalized treatment options. Eur J Hum Genet. 2024. 10.1038/s41431-024-01601-2 PMC11576736 38605127

[60] Rein B, Yan Z. 16p11.2 copy number variations and neurodevelopmental disorders. Trends Neurosci. 2020;43(11):886–901. 32993859 10.1016/j.tins.2020.09.001 PMC7606557

[61] Zinkstok JR, Boot E, Bassett AS, Hiroi N, Butcher NJ, Vingerhoets C, et al. Neurobiological perspective of 22q11.2 deletion syndrome. Lancet Psychiatry. 2019;6(11):951–60. 31395526 10.1016/S2215-0366(19)30076-8 PMC7008533

[62] Blagojevic C, Heung T, Theriault M, Tomita-Mitchell A, Chakraborty P, Kernohan K, et al. Estimate of the contemporary live-birth prevalence of recurrent 22q11.2 deletions: a cross-sectional analysis from population-based newborn screening. CMAJ Open. 2021;9(3):E802–9. 34404688 10.9778/cmajo.20200294 PMC8373039

[63] Bittel DC, Kibiryeva N, Sell SM, Strong TV, Butler MG. Whole genome microarray analysis of gene expression in Prader-Willi syndrome. Am J Med Genet A. 2007;143(5):430–42. 10.1002/ajmg.a.31606 PMC5467864 17236194

[64] Hogart A, Leung KN, Wang NJ, Wu DJ, Driscoll J, Vallero RO, et al. Chromosome 15q11-13 duplication syndrome brain reveals epigenetic alterations in gene expression not predicted from copy number. J Med Genet. 2009;46(2):86–93. 18835857 10.1136/jmg.2008.061580 PMC2634820

[65] Roden WH, Peugh LD, Jansen LA. Altered GABA(A) receptor subunit expression and pharmacology in human Angelman syndrome cortex. Neurosci Lett. 2010;483(3):167–72. 20692323 10.1016/j.neulet.2010.08.001 PMC3233535

[66] Samaco RC, Hogart A, LaSalle JM. Epigenetic overlap in autism-spectrum neurodevelopmental disorders: MECP2 deficiency causes reduced expression of UBE3A and GABRB3. Hum Mol Genet. 2005;14(4):483–92. 15615769 10.1093/hmg/ddi045 PMC1224722

[67] Scoles HA, Urraca N, Chadwick SW, Reiter LT, Lasalle JM. Increased copy number for methylated maternal 15q duplications leads to changes in gene and protein expression in human cortical samples. Molecular Autism. 2011;2:1–5. 22152151 10.1186/2040-2392-2-19 PMC3287113

[68] Borgatti R, Piccinelli P, Passoni D, Raggi E, Ferrarese C. Pervasive developmental disorders and GABAergic system in patients with inverted duplicated chromosome 15. J Child Neurol. 2001;16(12):911–4. 11785506 10.1177/088307380101601209

[69] Borgatti R, Piccinelli P, Passoni D, Romeo A, Viri M, Musumeci SA, et al. Peripheral markers of the gamma-aminobutyric acid (GABA) ergic system in Angelman’s syndrome. J Child Neurol. 2003;18:21–5. 12661934 10.1177/08830738030180010801

[70] Ebert MH, Schmidt DE, Thompson T, Butler MG. Elevated plasma gamma-aminobutyric acid (GABA) levels in individuals with either Prader-Willi syndrome or Angelman syndrome. J Neuropsychiatry Clin Neurosci. 1997;9(1):75–80. 9017532 10.1176/jnp.9.1.75 PMC5972534

[71] Evers LJ, van Amelsvoort TA, Bakker JA, de Koning M, Drukker M, Curfs LM. Glutamatergic markers, age, intellectual functioning and psychosis in 22q11 deletion syndrome. Psychopharmacology. 2015;232(18):3319–25. 26055684 10.1007/s00213-015-3979-x PMC4537490

[72] da Silva AF, Boot E, Schmitz N, Nederveen A, Vorstman J, Lavini C, et al. Proton magnetic resonance spectroscopy in 22q11 deletion syndrome. PLoS ONE. 2011;6(6):e21685. 21738766 10.1371/journal.pone.0021685 PMC3128078

[73] Mancini V, Saleh MG, Delavari F, Bagautdinova J, Eliez S. Excitatory/inhibitory imbalance underlies hippocampal atrophy in individuals with 22q11.2 deletion syndrome with psychotic symptoms. Biol Psychiatry. 2023;94(7):569–79. 37011759 10.1016/j.biopsych.2023.03.021

[74] Mori T, Mori K, Fujii E, Toda Y, Miyazaki M, Harada M, et al. Neuroradiological and neurofunctional examinations for patients with 22q11.2 deletion. Neuropediatrics. 2011;42(6):215–21. 22131192 10.1055/s-0031-1295479

[75] Rice LJ, Lagopoulos J, Brammer M, Einfeld SL. Reduced gamma-aminobutyric acid is associated with emotional and behavioral problems in Prader-Willi syndrome. Am J Med Genet B Neuropsychiatr Genet. 2016;171(8):1041–8. 27338833 10.1002/ajmg.b.32472

[76] Rogdaki M, Hathway P, Gudbrandsen M, McCutcheon RA, Jauhar S, et al. Glutamatergic function in a genetic high-risk group for psychosis: A proton magnetic resonance spectroscopy study in individuals with 22q11.2 deletion. Eur Neuropsychopharmacol. 2019;29(12):1333–42. 31648854 10.1016/j.euroneuro.2019.09.005

[77] van Hooijdonk CFM, Tse DHY, Roosenschoon J, Ceccarini J, Booij J, van Amelsvoort T, et al. The relationships between dopaminergic, glutamatergic, and cognitive functioning in 22q11.2 deletion syndrome: a cross-sectional, multimodal (1)H-MRS and (18)F-Fallypride PET study. Genes (Basel). 2022;13(9):1672. 36140839 10.3390/genes13091672 PMC9498700

[78] Vingerhoets C, Tse DH, van Oudenaren M, Hernaus D, van Duin E, Zinkstok J, et al. Glutamatergic and GABAergic reactivity and cognition in 22q11.2 deletion syndrome and healthy volunteers: a randomized double-blind 7-Tesla pharmacological MRS study. J Psychopharmacol. 2020;34(8):856–63. 32448020 10.1177/0269881120922977 PMC7376622

[79] Asahina N, Shiga T, Egawa K, Shiraishi H, Kohsaka S, Saitoh S. [^11^C]Flumazenil positron emission tomography analyses of brain gamma-aminobutyric acid type a receptors in angelman syndrome. J Pediatr. 2008;152:546-9.e3. 18346513 10.1016/j.jpeds.2007.08.038

[80] Holopainen IE, Metsahonkala EL, Kokkonen H, Parkkola RK, Manner TE, Nagren K, et al. Decreased binding of [^11^C]flumazenil in Angelman syndrome patients with GABA_A_ receptor beta_3_ subunit deletions. Ann Neurol. 2001;49:110–3. 11198279 10.1002/1531-8249(200101)49:1<110::aid-ana17>3.0.co;2-t

[81] Lucignani G, Panzacchi A, Bosio L, Moresco RM, Ravasi L, Coppa I, et al. GABA A receptor abnormalities in Prader-Willi syndrome assessed with positron emission tomography and [11C]flumazenil. Neuroimage. 2004;22(1):22–8. 15109994 10.1016/j.neuroimage.2003.10.050

[82] Donnelly NA, Bartsch U, Moulding HA, Eaton C, Marston H, Hall JH, et al. Sleep EEG in young people with 22q11.2 deletion syndrome: A cross-sectional study of slow-waves, spindles and correlations with memory and neurodevelopmental symptoms. Elife. 2022;11:e75482. 36039635 10.7554/eLife.75482 PMC9477499

[83] Egawa K, Saitoh S, Asahina N, Shiraishi H. Short-latency somatosensory-evoked potentials demonstrate cortical dysfunction in patients with Angelman syndrome. eNeurologicalSci. 2021;22:100298. 33313428 10.1016/j.ensci.2020.100298 PMC7721653

[84] Frohlich J, Miller MT, Bird LM, Garces P, Purtell H, Hoener MC, et al. Electrophysiological phenotype in angelman syndrome differs between genotypes. Biol Psychiatry. 2019;85(9):752–9. 30826071 10.1016/j.biopsych.2019.01.008 PMC6482952

[85] Frohlich J, Reiter LT, Saravanapandian V, DiStefano C, Huberty S, et al. Mechanisms underlying the EEG biomarker in Dup15q syndrome. Mol Autism. 2019;10:29. 31312421 10.1186/s13229-019-0280-6 PMC6609401

[86] Frohlich J, Senturk D, Saravanapandian V, Golshani P, Reiter LT, Sankar R, et al. A Quantitative electrophysiological biomarker of duplication 15q11.2-q13.1 syndrome. PLoS One. 2016;11(12):e0167179. 27977700 10.1371/journal.pone.0167179 PMC5157977

[87] Larsen KM, Pellegrino G, Birknow MR, Kjær TN, Baaré WFC, Didriksen M, et al. 22q11.2 deletion syndrome is associated with impaired auditory steady-state gamma response. Schizophr Bull. 2018;44(2):388–97. 28521049 10.1093/schbul/sbx058 PMC5815132

[88] Mancini V, Rochas V, Seeber M, Grent-’t-Jong T, Rihs TA, Latreche C, et al. Oscillatory neural signatures of visual perception across developmental stages in individuals with 22q11.2 deletion syndrome. Biol Psychiatry. 2022;92(5):407–18. 35550793 10.1016/j.biopsych.2022.02.961

[89] Mancini V, Rochas V, Seeber M, Roehri N, Rihs TA, Ferat V, et al. Aberrant developmental patterns of gamma-band response and long-range communication disruption in youths with 22q11.2 deletion syndrome. Am J Psychiatry. 2022;179(3):204–15. 35236117 10.1176/appi.ajp.2021.21020190

[90] Saravanapian V, Frohlich J, Hipp JF, Hyde C, Scheffler AW, Golshani P, et al. Properties of beta oscillations in Dup15q syndrome. J Neurodev Disord. 2020;12(1):22. 32791992 10.1186/s11689-020-09326-1 PMC7425173

[91] Saravanapian V, Nadkarni D, Hsu SH, Hussain SA, Maski K, Golshani P, et al. Abnormal sleep physiology in children with 15q11.2-13.1 duplication (Dup15q) syndrome. Mol Autism. 2021;12(1):54. 34344470 10.1186/s13229-021-00460-8 PMC8336244

[92] Dima DC, Adams R, Linden SC, Baird A, Smith J, Foley S, et al. Electrophysiological network alterations in adults with copy number variants associated with high neurodevelopmental risk. Transl Psychiatry. 2020;10(1):324. 32958742 10.1038/s41398-020-00998-w PMC7506525

[93] Doherty JL, Cunningham AC, Chawner S, Moss HM, Dima DC, Linden DEJ, et al. Atypical cortical networks in children at high-genetic risk of psychiatric and neurodevelopmental disorders. Neuropsychopharmacology. 2024;49(2):368–76. 37402765 10.1038/s41386-023-01628-x PMC7615386

[94] Egawa K, Asahina N, Shiraishi H, Kamada K, Takeuchi F, Nakane S, et al. Aberrant somatosensory-evoked responses imply GABAergic dysfunction in Angelman syndrome. Neuroimage. 2008;39(2):593–9. 17962046 10.1016/j.neuroimage.2007.09.006

[95] Egawa K, Saitoh S, Asahina N, Shiraishi H. Variance in the pathophysiological impact of the hemizygosity of gamma-aminobutyric acid type A receptor subunit genes between Prader-Willi syndrome and Angelman syndrome. Brain Dev. 2021;43(4):521–7. 33419637 10.1016/j.braindev.2020.12.014

[96] Avazzadeh S, McDonagh K, Reilly J, Wang Y, Boomkamp SD, McInerney V, et al. Increased Ca2+ signaling in NRXN1α +/- neurons derived from ASD induced pluripotent stem cells. Mol Autism. 2019;10(1):1–6. 31893021 10.1186/s13229-019-0303-3 PMC6937972

[97] Avazzadeh S, Quinlan LR, Reilly J, McDonagh K, Jalali A, Wang Y, et al. NRXN1α(+/-) is associated with increased excitability in ASD iPSC-derived neurons. BMC Neurosci. 2021;22(1):56. 34525970 10.1186/s12868-021-00661-0 PMC8442436

[98] Fink JJ, Robinson TM, Germain ND, Sirois CL, Bolduc KA, Ward AJ, et al. Disrupted neuronal maturation in Angelman syndrome-derived induced pluripotent stem cells. Nat Commun. 2017;8:15038. 28436452 10.1038/ncomms15038 PMC5413969

[99] Fink JJ, Schreiner JD, Bloom JE, James J, Baker DS, Robinson TM, et al. Hyperexcitable phenotypes in induced pluripotent stem cell-derived neurons from patients with 15q11-q13 duplication syndrome, a genetic form of autism. Biol Psychiatry. 2021;90(11):756–65. 34538422 10.1016/j.biopsych.2021.07.018 PMC8571044

[100] Hussein Y, Tripathi U, Choudhary A, Nayak R, Peles D, Rosh I, et al. Early maturation and hyperexcitability is a shared phenotype of cortical neurons derived from different ASD-associated mutations. Transl Psychiatry. 2023;13(1):246. 37414777 10.1038/s41398-023-02535-x PMC10326262

[101] Khan TA, Revah O, Gordon A, Yoon SJ, Krawisz AK, Goold C, et al. Neuronal defects in a human cellular model of 22q11.2 deletion syndrome. Nat Med. 2020;26(12):1888–98. 32989314 10.1038/s41591-020-1043-9 PMC8525897

[102] Khattak S, Brimble E, Zhang W, Zaslavsky K, Strong E, Ross PJ, et al. Human induced pluripotent stem cell derived neurons as a model for Williams-Beuren syndrome. Mol Brain. 2015;8(1):77. 26603386 10.1186/s13041-015-0168-0 PMC4657290

[103] Meganathan K, Prakasam R, Baldridge D, Gontarz P, Zhang B, Urano F, et al. Altered neuronal physiology, development, and function associated with a common chromosome 15 duplication involving CHRNA7. BMC Biol. 2021;19(1):147. 34320968 10.1186/s12915-021-01080-7 PMC8317352

[104] Nehme R, Pietilainen O, Artomov M, Tegtmeyer M, Valakh V, Lehtonen L, et al. The 22q11.2 region regulates presynaptic gene-products linked to schizophrenia. Nat Commun. 2022;13(1):3690. 35760976 10.1038/s41467-022-31436-8 PMC9237031

[105] Pak C, Danko T, Mirabella VR, Wang J, Liu Y, Vangipuram M, et al. Cross-platform validation of neurotransmitter release impairments in schizophrenia patient-derived NRXN1-mutant neuron. Proc Natl Acad Sci U S A. 2021;118(22):e2025598118. 34035170 10.1073/pnas.2025598118 PMC8179243

[106] Pak C, Danko T, Zhang Y, Aoto J, Anderson G, Maxeiner S, et al. Human neuropsychiatric disease modeling using conditional deletion reveals synaptic transmission defects caused by heterozygous mutations in NRXN1. Cell Stem Cell. 2015;17(3):316–28. 26279266 10.1016/j.stem.2015.07.017 PMC4560990

[107] Parnell E, Culotta L, Forrest MP, Jalloul HA, Eckman BL, Loizzo DD, et al. excitatory dysfunction drives network and calcium handling deficits in 16p11.2 duplication schizophrenia induced pluripotent stem cell-derived neurons. Biol Psychiatry. 2023;94(2):153–63. 36581494 10.1016/j.biopsych.2022.11.005 PMC10166768

[108] Sebastian R, Jin K, Pavon N, Bansal R, Potter A, Song Y, et al. Schizophrenia-associated NRXN1 deletions induce developmental-timing- and cell-type-specific vulnerabilities in human brain organoids. Nat Commun. 2023;14(1):3770. 37355690 10.1038/s41467-023-39420-6 PMC10290702

[109] Tai DJC, Razaz P, Erdin S, Gao D, Wang J, Nuttle X, et al. Tissue- and cell-type-specific molecular and functional signatures of 16p11.2 reciprocal genomic disorder across mouse brain and human neuronal models. Am J Hum Genet. 2022;109(10):1789–813. 36152629 10.1016/j.ajhg.2022.08.012 PMC9606388

[110] Zhao D, Lin M, Chen J, Pedrosa E, Hrabovsky A, Fourcade HM, et al. MicroRNA profiling of neurons generated using induced pluripotent stem cells derived from patients with schizophrenia and schizoaffective disorder, and 22q11.2 Del. PLoS One. 2015;10(7):e0132387. 26173148 10.1371/journal.pone.0132387 PMC4501820

[111] Choi YB, Mentch J, Haskins AJ, Van Wicklin C, Robertson CE. Visual processing in genetic conditions linked to autism: a behavioral study of binocular rivalry in individuals with 16p11.2 deletions and age-matched controls. Autism Res. 2023;16(4):831–40. 36751102 10.1002/aur.2901

[112] Bird LM, Ochoa-Lubinoff C, Tan WH, Heimer G, Melmed RD, Rakhit A, et al. The STARS phase 2 study: a randomized controlled trial of gaboxadol in angelman syndrome. Neurology. 2021;96:E1024–35. 33443117 10.1212/WNL.0000000000011409 PMC8055330

[113] Keary C, Bird LM, de Wit MC, Hatti S, Heimer G, Heussler H, et al. Gaboxadol in angelman syndrome: a double-blind, parallel-group, randomized placebo-controlled phase 3 study. Eur J Paediatr Neurol. 2023;47:6–12. 37639777 10.1016/j.ejpn.2023.07.008

[114] Civardi C, Vicentini R, Grugni G, Cantello R. Corticospinal physiology in patients with Prader-Willi syndrome: a transcranial magnetic stimulation study. Arch Neurol. 2004;61(10):1585–9. 15477513 10.1001/archneur.61.10.1585

[115] Hawkins RA. The blood-brain barrier and glutamate. Am J Clin Nutr. 2009;90(3):867S-S874. 19571220 10.3945/ajcn.2009.27462BB PMC3136011

[116] Kakee A, Takanaga H, Terasaki T, Naito M, Tsuruo T, Sugiyama Y. Efflux of a suppressive neurotransmitter, GABA, across the blood-brain barrier. J Neurochem. 2001;79(1):110–8. 11595763 10.1046/j.1471-4159.2001.00540.x

[117] Majo VJ, Prabhakaran J, Mann JJ, Kumar JS. PET and SPECT tracers for glutamate receptors. Drug Discov Today. 2013;18(3–4):173–84. 23092894 10.1016/j.drudis.2012.10.004

[118] Andersson JD, Matuskey D, Finnema SJ. Positron emission tomography imaging of the gamma-aminobutyric acid system. Neurosci Lett. 2019;691:35–43. 30102960 10.1016/j.neulet.2018.08.010

[119] Ahmad J, Ellis C, Leech R, Voytek B, Garces P, Jones E, et al. From mechanisms to markers: novel noninvasive EEG proxy markers of the neural excitation and inhibition system in humans. Transl Psychiatry. 2022;12(1):467. 36344497 10.1038/s41398-022-02218-z PMC9640647

[120] Drakulic D, Djurovic S, Syed YA, Trattaro S, Caporale N, Falk A, et al. Copy number variants (CNVs): a powerful tool for iPSC-based modelling of ASD. Molecular Autism. 2020;11(1):1–8. 32487215 10.1186/s13229-020-00343-4 PMC7268297

[121] Hoffmann A, Ziller M, Spengler D. Childhood-onset schizophrenia: insights from induced pluripotent stem cells. Int J Mol Sci. 2018;19(12):3829. 30513688 10.3390/ijms19123829 PMC6321410

[122] Dori N, Green T, Weizman A, Gothelf D. The effectiveness and safety of antipsychotic and antidepressant medications in individuals with 22q11.2 deletion syndrome. J Child Adolesc Psychopharmacol. 2017;27(1):83–90. 26131914 10.1089/cap.2014.0075

[123] Tang SX, Yi JJ, Calkins ME, Whinna DA, Kohler CG, Souders MC, et al. Psychiatric disorders in 22q11.2 deletion syndrome are prevalent but undertreated. Psychol Med. 2014;44(6):1267–77. 24016317 10.1017/S0033291713001669 PMC4461220

[124] Wafford KA, Ebert B. Gaboxadol–a new awakening in sleep. Curr Opin Pharmacol. 2006;6(1):30–6. 16368265 10.1016/j.coph.2005.10.004

[125] Doble A. The pharmacology and mechanism of action of riluzole. Neurology. 1996;47:S233–41. 8959995 10.1212/wnl.47.6_suppl_4.233s

[126] Feld GB, Born J. Neurochemical mechanisms for memory processing during sleep: basic findings in humans and neuropsychiatric implications. Neuropsychopharmacology. 2020;45(1):31–44. 31443105 10.1038/s41386-019-0490-9 PMC6879745

[127] Uzunova G, Pallanti S, Hollander E. Excitatory/inhibitory imbalance in autism spectrum disorders: Implications for interventions and therapeutics. World J Biol Psychiatry. 2016;17(3):174–86. 26469219 10.3109/15622975.2015.1085597

[128] Owen MJ, O’Donovan MC. Schizophrenia and the neurodevelopmental continuum: evidence from genomics. World Psychiatry. 2017;16(3):227–35. 28941101 10.1002/wps.20440 PMC5608820

[129] Mosheva M, Korotkin L, Gur RE, Weizman A, Gothelf D. Effectiveness and side effects of psychopharmacotherapy in individuals with 22q11.2 deletion syndrome with comorbid psychiatric disorders: a systematic review. Eur Child Adolesc Psychiatry. 2020;29(8):1035–48. 30949827 10.1007/s00787-019-01326-4

[130] Ghodke-Puranik Y, Thorn CF, Lamba JK, Leeder JS, Song W, Birnbaum AK, et al. Valproic acid pathway: pharmacokinetics and pharmacodynamics. Pharmacogenet Genomics. 2013;23(4):236–41. 23407051 10.1097/FPC.0b013e32835ea0b2 PMC3696515

[131] Vingerhoets C, Tse DHY, van Amelsvoort T. Riluzole effectively treats psychotic symptoms and improves cognition in 22q11.2 deletion syndrome: a clinical case. Eur J Med Genet. 2019;62(8):103705. 31229682 10.1016/j.ejmg.2019.103705

[132] Farokhnia M, Sabzabadi M, Pourmahmoud H, Khodaie-Ardakani MR, Hosseini SM, Yekehtaz H, et al. A double-blind, placebo controlled, randomized trial of riluzole as an adjunct to risperidone for treatment of negative symptoms in patients with chronic schizophrenia. Psychopharmacology. 2014;231(3):533–42. 24013610 10.1007/s00213-013-3261-z

[133] Vingerhoets C, Sylvester A, Tse DH, Serrarens C, Janssen P, Swillen A, et al. Riluzole as Cognitive Enhancer in 22q11.2 Deletion Syndrome? The 13th Biennial International 22q112 Scientific Meeting; 18/07/2024; Obidos, Portugal2024.

[134] Elia J, Ungal G, Kao C, Ambrosini A, De Jesus-Rosario N, Larsen L, et al. Fasoracetam in adolescents with ADHD and glutamatergic gene network variants disrupting mGluR neurotransmitter signaling. Nat Commun. 2018;9(1):4. 29339723 10.1038/s41467-017-02244-2 PMC5770454

[135] Chadehumbe M, Hopkins S, Gur RC, McDonald-McGinn D, Gallagher ER, Conant KD, et al. A Randomized, Double-blind, Placebo-controlled Phase 2 Clinical Trial of NB-001 (fasoracetam) for Neuropsychiatric Symptoms in Children and Adolescents with 22q11 Deletion Syndrome (22q11DS) The 13th Biennial International 22q112 Scientific Meeting; 18/07/2024; Obidos, Portugal2024.

[136] Finisguerra A, Borgatti R, Urgesi C. Non-invasive brain stimulation for the rehabilitation of children and adolescents with neurodevelopmental disorders: a systematic review. Front Psychol. 2019;10:135. 30787895 10.3389/fpsyg.2019.00135 PMC6373438

[137] Latreche C, Mancini V, Rochas V, Maeder J, Cantonas LM, Ferat V, et al. Using transcranial alternating current stimulation to enhance working memory skills in youths with 22q11.2 deletion syndrome: a randomized double-blind sham-controlled study. Psychiatry Res. 2024;335:115835. 38460352 10.1016/j.psychres.2024.115835

[138] Chawner SJ, Watson CJ, Owen MJ. Clinical evaluation of patients with a neuropsychiatric risk copy number variant. Curr Opin Genet Dev. 2021;68:26–34. 33461126 10.1016/j.gde.2020.12.012 PMC8219523

[139] Gonzalez-Castro TB, Hernandez-Diaz Y, Juarez-Rojop IE, Lopez-Narvaez ML, Tovilla-Zarate CA, Fresan A. The Role of a Catechol-O-Methyltransferase (COMT) Val158Met genetic polymorphism in schizophrenia: a systematic review and updated meta-analysis on 32,816 subjects. Neuromolecular Med. 2016;18(2):216–31. 27020768 10.1007/s12017-016-8392-z

[140] Lopez SJ, Segal DJ, LaSalle JM. UBE3A: An E3 ubiquitin ligase with genome-wide impact in neurodevelopmental disease. Front Mol Neurosci. 2018;11:476. 30686997 10.3389/fnmol.2018.00476 PMC6338038

[141] Schizophrenia Working Group of the Psychiatric Genomics Consortium. Biological insights from 108 schizophrenia-associated genetic loci. Nature. 2014;511(7510):421–7. 25056061 10.1038/nature13595 PMC4112379

[142] Autism Spectrum Disorders Working Group of The Psychiatric Genomics Consortium. Meta-analysis of GWAS of over 16,000 individuals with autism spectrum disorder highlights a novel locus at 10q24.32 and a significant overlap with schizophrenia. Mol Autism. 2017;8:21. 10.1186/s13229-017-0137-9 PMC5441062 28540026

[143] Elia J, Glessner JT, Wang K, Takahashi N, Shtir CJ, Hadley D, et al. Genome-wide copy number variation study associates metabotropic glutamate receptor gene networks with attention deficit hyperactivity disorder. Nat Genet. 2011;44(1):78–84. 22138692 10.1038/ng.1013 PMC4310555

[144] Al-Absi AR, Qvist P, Okujeni S, Khan AR, Glerup S, Sanchez C, et al. Layers II/III of prefrontal cortex in Df(h22q11)/+ mouse model of the 22q11.2 deletion display loss of parvalbumin interneurons and modulation of neuronal morphology and excitability. Mol Neurobiol. 2020;57(12):4978–88. 32820460 10.1007/s12035-020-02067-1

[145] Al-Absi AR, Thambiappa SK, Khan AR, Glerup S, Sanchez C, Landau AM, et al. Df(h22q11)/+ mouse model exhibits reduced binding levels of GABA(A) receptors and structural and functional dysregulation in the inhibitory and excitatory networks of hippocampus. Mol Cell Neurosci. 2022;122:103769. 35988854 10.1016/j.mcn.2022.103769

[146] Silva AI, Ehrhart F, Ulfarsson MO, Stefansson H, Stefansson K, Wilkinson LS, et al. Neuroimaging findings in neurodevelopmental copy number variants: identifying molecular pathways to convergent phenotypes. Biol Psychiatry. 2022;92(5):341–61. 35659384 10.1016/j.biopsych.2022.03.018

[147] Pasanta D, He JL, Ford T, Oeltzschner G, Lythgoe DJ, Puts NA. Functional MRS studies of GABA and glutamate/Glx - a systematic review and meta-analysis. Neurosci Biobehav Rev. 2023;144:104940. 36332780 10.1016/j.neubiorev.2022.104940 PMC9846867

[148] Taylor R, Neufeld RW, Schaefer B, Densmore M, Rajakumar N, Osuch EA, et al. Functional magnetic resonance spectroscopy of glutamate in schizophrenia and major depressive disorder: anterior cingulate activity during a color-word Stroop task. NPJ Schizophr. 2015;1:15028. 27336037 10.1038/npjschz.2015.28 PMC4849454

[149] Jelen LA, King S, Horne CM, Lythgoe DJ, Young AH, Stone JM. Functional magnetic resonance spectroscopy in patients with schizophrenia and bipolar affective disorder: Glutamate dynamics in the anterior cingulate cortex during a working memory task. Eur Neuropsychopharmacol. 2019;29(2):222–34. 30558824 10.1016/j.euroneuro.2018.12.005

[150] Stagg CJ, Bachtiar V, Amadi U, Gudberg CA, Ilie AS, Sampaio-Baptista C, et al. Local GABA concentration is related to network-level resting functional connectivity. Elife. 2014;3:e01465. 24668166 10.7554/eLife.01465 PMC3964822

[151] Duncan NW, Wiebking C, Tiret B, Marjanska M, Hayes DJ, Lyttleton O, et al. Glutamate concentration in the medial prefrontal cortex predicts resting-state cortical-subcortical functional connectivity in humans. PLoS ONE. 2013;8(4):e60312. 23573246 10.1371/journal.pone.0060312 PMC3616113

[152] Maximo JO, Briend F, Armstrong WP, Kraguljac NV, Lahti AC. Salience network glutamate and brain connectivity in medication-naive first episode patients - a multimodal magnetic resonance spectroscopy and resting state functional connectivity MRI study. Neuroimage Clin. 2021;32:102845. 34662778 10.1016/j.nicl.2021.102845 PMC8526757

[153] Morrison S, Chawner S, van Amelsvoort T, Swillen A, Vingerhoets C, Vergaelen E, et al. Cognitive deficits in childhood, adolescence and adulthood in 22q11.2 deletion syndrome and association with psychopathology. Transl Psychiatry. 2020;10(1):53. 32066691 10.1038/s41398-020-0736-7 PMC7026075

[154] Schneider M, Debbane M, Bassett AS, Chow EW, Fung WL, van den Bree M, et al. Psychiatric disorders from childhood to adulthood in 22q11.2 deletion syndrome: results from the international consortium on brain and behavior in 22q11.2 deletion syndrome. Am J Psychiatry. 2014;171(6):627–39. 24577245 10.1176/appi.ajp.2013.13070864 PMC4285461

[155] Bernier R, Hudac CM, Chen Q, Zeng C, Wallace AS, Gerdts J, et al. Developmental trajectories for young children with 16p11.2 copy number variation. Am J Med Genet B Neuropsychiatr Genet. 2017;174(4):367–80. 28349640 10.1002/ajmg.b.32525

[156] Deep-Soboslay A, Benes FM, Haroutunian V, Ellis JK, Kleinman JE, Hyde TM. Psychiatric brain banking: three perspectives on current trends and future directions. Biol Psychiatry. 2011;69(2):104–12. 20673875 10.1016/j.biopsych.2010.05.025 PMC3105380

