# Supplementary material for: Neural excitation/inhibition imbalance and neurodevelopmental pathology in human copy number variant syndromes: a systematic review
Source: J Neurodev Disord. 2025 Jun 9;17:31. doi: 10.1186/s11689-025-09614-8 (PMC12147258; doi:10.1186/s11689-025-09614-8)
Supplement: Supplementary file 1 — Supplementary Material 1. [file 11689_2025_9614_MOESM1_ESM.docx]

**Supplement 1. Search strategy**

15q11.2 OR 15q13.3 OR 7q11.23 OR 22q11.2 OR 22q11 OR 1q21.1 OR 3q29 OR 16p11.2 OR 16p13.11 OR 17p12 OR 17q12 OR 2p16.3 OR williams beuren OR velocardiofacial OR angelman OR deletion syndrome OR duplication syndrome OR chromosome deletion OR chromosome duplication OR digeorge OR copy number varia* OR CNV OR prader willi OR genetic high risk OR gene deletion OR gene duplication OR microdeletion OR microduplication OR shprintzen

AND

excitat* OR inhibit* OR e i balance OR e i imbalance OR GABA OR gamma aminobutyric acid OR GABAergic OR glutamat* OR Glu OR Glx OR glutamine OR Gln OR ePSP OR iPSP OR post synap* OR postsynap* OR proline OR PRODH OR AMPA OR AMPAR OR ampa receptor OR NMDA OR NMDAR OR nmda receptor OR N-methyl-D-aspartate OR n methyl d aspartate receptor OR metabotropic OR mGlu OR kain* OR ionotropic

AND

attention OR cogniti* OR memory OR intelligence OR IQ OR intellectual disability OR mental retardation OR learning disability OR ADHD OR ADD OR attention deficit OR hyperactiv* OR inattenti* OR neurodevelop* OR autis* OR ASD OR ASC OR asperger* OR pervasive developmental disorder OR PDD OR psychosis OR psychotic OR schizoaffect* OR schizophren* OR schizotyp* OR bipolar OR manic depressi* OR mania
